# Molecular network of metabolic reprogramming and precision diagnosis and treatment of hepatocellular carcinoma

**DOI:** 10.1186/s40364-025-00844-5

**Published:** 2025-10-10

**Authors:** Lingbo An, Zongfang Li

**Affiliations:** 1 Department of General Surgery, National-Local Joint Engineering Research Center of Biodiagnostic & Biotherapy, The Second Affiliated Hospital of Xi’an Jiaotong University, Xi’an, 710004 Shaanxi China; 2 Shaanxi Provincial Clinical Medical Research Center for Liver and Spleen Diseases, CHESS-Shaanxi consortium, The Second Affiliated Hospital of Xi’an Jiaotong University, Xi’an, 710004 Shaanxi China; 3 Inflammation and Immunity International Science and Technology Cooperation Base of Shaanxi Province, Xi’an, 710038 Shaanxi China; 4 Key Laboratory of Environment and Genes Related to Diseases, Xi’an Jiaotong University, Ministry of Education of China, Xi’an, 710061 Shaanxi China; 5 Department of General Surgery, National-Local Joint Engineering Research Center of Biodiagnostic & Biotherapy, Shaanxi Provincial Clinical Medical Research Center for Liver and Spleen Diseases, Key Laboratory of Environment and Genes Related to Diseases, CHESS-Shaanxi consortium, Shaanxi International Cooperation Base for Inflammation and Immunity, Shaanxi Provincial Academician Workstation, The Second Affiliated Hospital of Xi’an Jiaotong University, Xi’an Jiaotong University, Xi’an, China

**Keywords:** Hepatocellular carcinoma, Metabolic reprogramming, Metabolic networks, Metabolic–signaling interactions, Targeted drugs, Combined diagnosis and treatment

## Abstract

Primary liver cancer, particularly hepatocellular carcinoma (HCC), remains a major cause of cancer-related mortality worldwide, with rising incidence and limited treatment options, especially for patients diagnosed at advanced stages. In recent years, metabolic reprogramming has emerged as a hallmark of cancer that enables HCC cells to survive, proliferate, and resist therapy under hostile conditions. HCC cells undergo profound remodeling of glucose, lipid, and amino acid metabolism to adapt to hypoxia and nutrient deprivation. These processes are orchestrated by key signaling cascades, including the PI3K/AKT/mTOR, Ras-Raf-MEK-ERK-cMYC, and LKB1-AMPK pathways, forming a dynamic and integrated metabolic-signaling network. This review comprehensively integrates recent advances in the understanding of metabolic pathways in HCC, with a particular focus on glycolysis, de novo lipogenesis, and glutamine metabolism. We delineate the regulatory mechanisms underlying these pathways and construct an interaction map linking metabolic circuits to clinical phenotypes such as tumor heterogeneity, metastatic potential, and immune modulation. Furthermore, we systematically evaluate the biomarker potential of metabolic intermediates, rate-limiting enzymes, and key regulators in the context of early detection, molecular classification, prognosis prediction, and therapeutic response in HCC. We also highlight cutting-edge technologies, including metabolic imaging, liquid biopsy-based biomarker detection, and metabolism-targeted therapies. The review explores their potential synergy with immunotherapy, chemotherapy, and radiotherapy, aiming to provide a comprehensive framework for individualized HCC management. Our discussion underscores the translational relevance of metabolic biomarkers and offers insights for future research and clinical innovation.

## Introduction

**Fig. 1:**
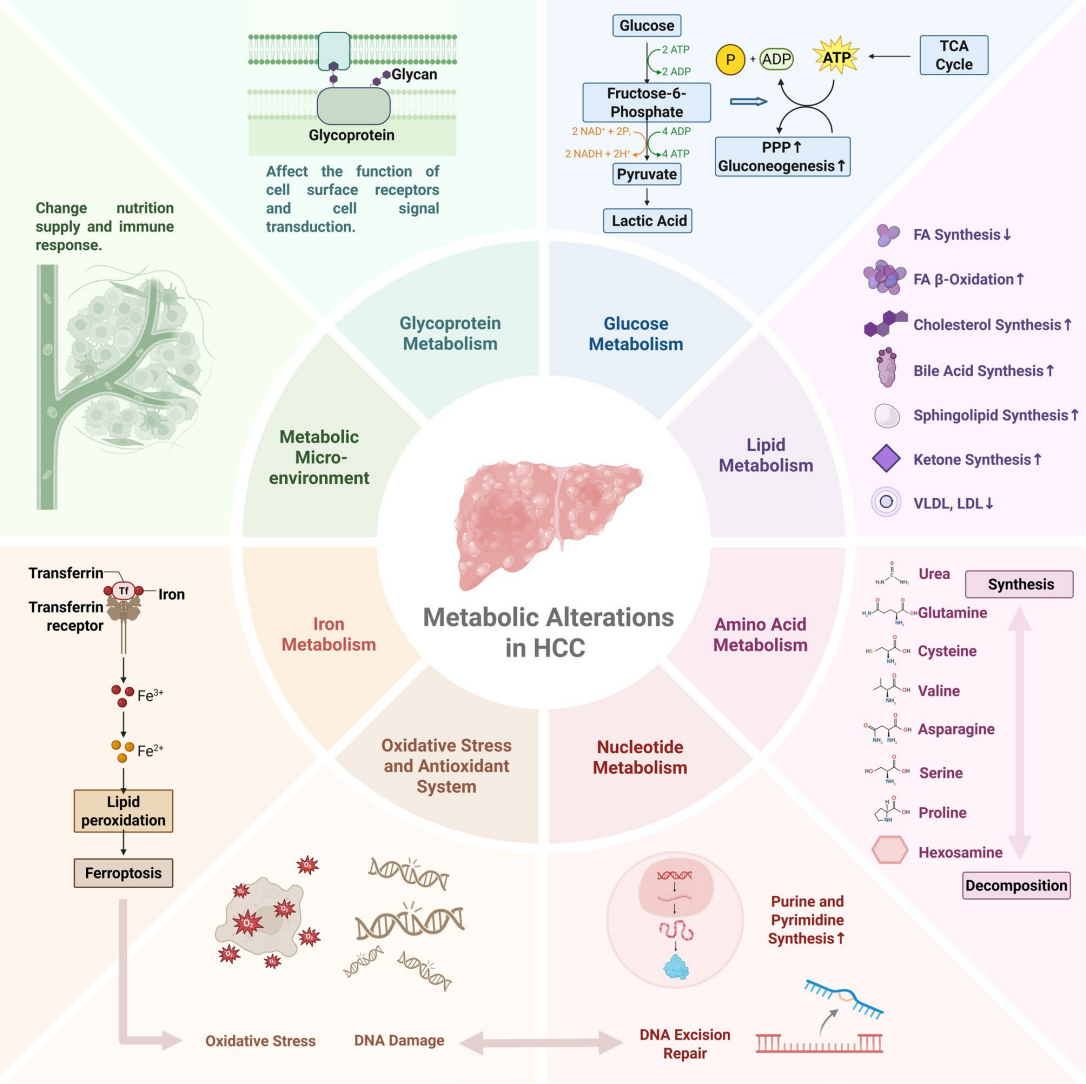
Metabolic alternations in HCC. Metabolic dysregulation is emphasized in the pathogenesis of HCC, HIF-mediated hypoxia can induce abnormal gene expression of most relevant enzymes in the HCC metabolic environment. Lipids and their degradation pathways regulate ferroptosis by modulating intracellular levels of lipid peroxides, glutathione, malondialdehyde and Fe^2+^. Ferroptosis can further induce DNA damage and oxidative stress. VLDL, very low-density lipoproteins; LDL, low-density lipoproteins. Created with BioRender.com

Primary liver cancer ranks as the sixth most common cancer and the third leading cause of cancer-related mortality worldwide, with hepatocellular carcinoma (HCC) accounting for 75–85% of primary liver cancer cases [1]. Among newly diagnosed HCC cases globally, up to 71% occur in the Asia-Pacific region, with 42% concentrated in China [1]. The transition from normal cells to malignant tumor phenotypes is accompanied by significant alterations in the tumor microenvironment (TME), where rapid adaptive responses to hypoxia and nutrient deprivation are collectively termed “metabolic reprogramming”. The concept of cancer metabolic reprogramming traces back to Warburg, who observed that tumor tissues preferentially rely on glycolysis for energy production even under aerobic conditions [2]. As research progressed, the focus of cancer metabolism expanded beyond glucose metabolism to lipids, amino acids, and other metabolic pathways. Systems biology-based analyses of metabolic network dependencies have provided comprehensive insights into the metabolic alterations occurring in HCC (Fig. 1).

The molecular network of metabolic reprogramming in HCC refers to the systematic modulation of metabolic pathways driven by genetic mutations, epigenetic modifications, and activation of signaling pathways, enabling HCC cells to sustain rapid proliferation, invasion, and resistance to adverse conditions. Within the complex tumor microenvironment, metabolic reprogramming in HCC is closely associated with activation of the PI3K/AKT/mTOR and rat sarcoma virus (Ras) / rapidly accelerated fibrosarcoma (Raf) / mitogen-activated protein kinase kinase (MEK) / extracellular signal-regulated kinase (ERK) / c-Myc (Ras-Raf-MEK-ERK-cMYC) pathways, as well as inactivation of the liver kinase B1 / AMP-activated protein kinase (LKB1-AMPK) pathway. These regulatory mechanisms are fundamental to HCC cell survival and proliferation. Among them, the PI3K/AKT/mTOR pathway serves as the central metabolic regulator, not only supporting HCC proliferation by promoting glucose, lipid, and protein metabolism but also optimizing metabolic efficiency through transcriptional and translational regulation of metabolic enzymes.

Existing therapeutic strategies, such as small-molecule tyrosine kinase inhibitors (TKIs) or monoclonal antibodies (primarily sorafenib, lenvatinib, and ramucirumab), provide only limited overall survival benefits and are associated with severe adverse effects, including hand-foot skin reactions and myocardial ischemia [3]. Although immune checkpoint inhibitors have demonstrated significant improvements in median overall survival (OS), progression-free survival (PFS), and objective response rates (ORR), most patients with advanced HCC still exhibit chemoresistance, posing new challenges in HCC therapy. Given that dysregulated metabolic networks are key molecular mechanisms underlying HCC resistance and that conventional therapies fail to achieve optimal patient outcomes, addressing the following critical questions may help elucidate the pathogenesis of HCC, identify specific diagnostic biomarkers, and determine therapeutic targets, ultimately facilitating precision diagnosis and treatment targeting metabolic pathways in HCC. What metabolic mechanisms specifically accommodate the unique growth requirements of HCC cells? Do crosstalk mechanisms exist among different metabolic pathways, and if so, how do they function? How does metabolic reprogramming promote HCC cell proliferation, invasion, and metastasis? Most importantly, what metabolic-targeted drugs can be developed against these pathways, and what are their specific pharmacological mechanisms? Can they be combined with immunotherapy, chemotherapy, or radiotherapy? What are the therapeutic outcomes?

## Molecular network of metabolic reprogramming in HCC

**Fig. 2:**
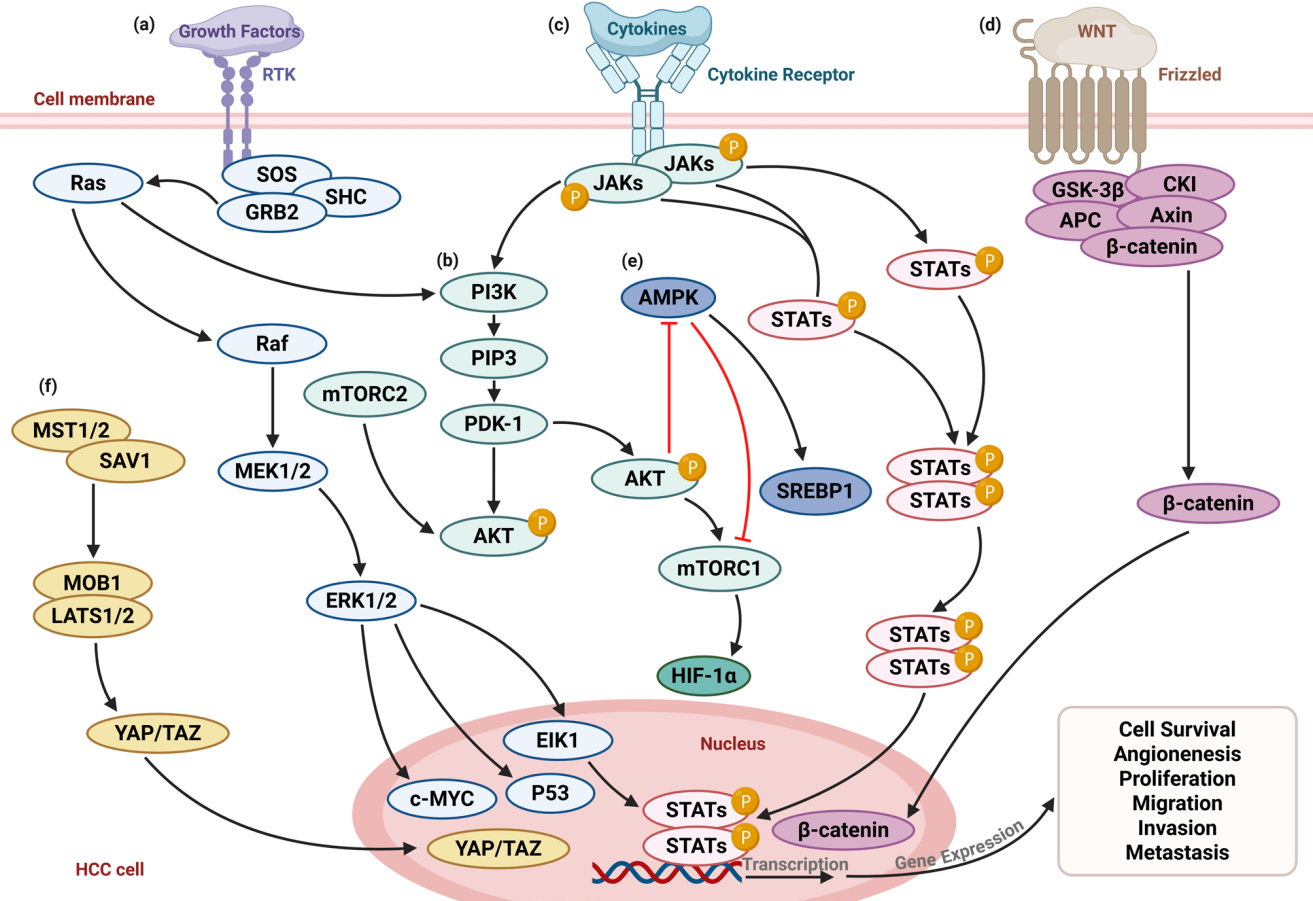
Key signaling pathways/axes involved in metabolism reprogramming of HCC. Metabolic reprogramming in HCC is the result of a complex molecular network driven by multiple signaling pathways. Key signaling pathways/axes, including (**a**) the Ras/Raf/MEK/ERK pathway, (**b**) the PI3K/AKT/mTOR pathway, (**c**) the JAK/STAT pathway, (**d**) the Wnt/β-catenin pathway, (**e**) the AMPK–SREBP1 axis, and (**f**) the Hippo–YAP/TAZ pathway, collaboratively regulate glucose metabolism, lipid metabolism, amino acid metabolism, and redox balance, thereby shaping a metabolic microenvironment that favors HCC cell adaptation to hypoxia, proliferative demands, and immune evasion. Created with BioRender.com

In HCC, metabolic reprogramming serves as a core feature of HCC cell adaptive survival and represents a downstream effect collectively regulated by multiple oncogenic signaling pathways. These pathways reshape the metabolic networks of glucose, lipids, amino acids, and more in HCC cells by modulating key metabolic enzymes, transcription factors, and metabolite transporters, thereby establishing a metabolic microenvironment conducive to HCC progression. The roles of several key signaling pathways/axes in HCC are as follows: (i) Ras/Raf/MEK/ERK pathway: This cascade is activated downstream of membrane-bound receptor tyrosine kinases (RTKs) upon growth factor stimulation, leading to phosphorylation of Ras, Raf, MEK, and ERK. ERK translocates to the nucleus and promotes transcription of key regulators, including MYC and EIK1, which enhance glycolytic gene expression. Ras also cross-activates the PI3K pathway, illustrating its role as a central node linking proliferation and metabolic control [4]. (ii) PI3K/AKT/mTOR pathway: A pivotal metabolic axis, PI3K phosphorylates PIP2 to generate PIP3, which recruits and activates PDK1 and AKT. Activated AKT enhances glucose uptake, lipid biosynthesis, and inhibits apoptosis by activating mTORC1. mTORC1 promotes anabolic metabolism via HIF-1α and SREBP1, while AKT suppresses AMPK [5]. (iii) JAK/STAT pathway: Inflammatory cytokines activate Janus kinases (JAKs), which phosphorylate STAT3. Phospho-STAT3 dimerizes and translocates to the nucleus, promoting transcription of genes involved in proliferation, anti-apoptosis, and immune evasion. STAT3 also regulates metabolic enzymes, linking chronic inflammation to metabolic reprogramming [6]. (iv) Wnt/β-catenin pathway: Activation of Wnt ligands stabilize β-catenin, allowing its nuclear accumulation and interaction with TCF/LEF transcription factors. This enhances expression of cyclin D1 and metabolic genes involved in glutamine metabolism and lipid synthesis, contributing to HCC cell proliferation and metabolic adaptation [7]. (v) AMPK–SREBP1 axis: AMPK acts as an energy sensor that inhibits anabolic processes under metabolic stress. When activated, it suppresses lipid biosynthesis by inhibiting SREBP1 maturation and activity. Conversely, in HCC, decreased AMPK activity or increased AKT signaling facilitates SREBP1-driven lipid accumulation, supporting membrane synthesis and HCC growth. mTOR is a crucial downstream modulator of AMPK signaling in HCC cells. AMPK is a key energy stress sensor that antagonizes mTORC1 activity [8, 9]. (vi) Hippo–YAP/TAZ pathway: This pathway controls cell fate and organ size. The MST1/2–SAV1 kinase complex activates LATS1/2, which phosphorylates and inhibits the transcription coactivators YAP/TAZ. In HCC, Hippo pathway inactivation leads to YAP/TAZ nuclear translocation, where they drive transcription of genes promoting glycolysis, glutamine metabolism, and proliferation, linking mechanotransduction to metabolic rewiring [10]. Figure 2 summarizes the key signaling pathways/axes involved in the metabolic reprogramming of HCC described above. In addition, the metabolic network of HCC cells exhibits high plasticity, with extensive crosstalk among metabolic pathways. The underlying mechanisms of this inter-pathway interaction will be discussed in detail in Sect. 2. In the following sections, we will focus on glucose metabolism, lipid metabolism, and amino acid metabolism in HCC to illustrate the molecular landscape of metabolic reprogramming and highlight the latest research findings.

### Key molecular network of glucose metabolic reprogramming

#### Glycolytic pathway

Glycolysis, the process by which glucose is broken down into pyruvate in the cytoplasm, during which two molecules of pyruvate and two molecules of ATP are produced for every molecule of glucose broken down, serves as the initial step for both anaerobic and aerobic glucose metabolism. This pathway is tightly regulated by three rate-limiting enzymes: hexokinase (HK), phosphofructokinase-1 (PFK), and pyruvate kinase (PKM). HK, the enzyme catalyzing the first step of glycolysis, has four isoforms (HK1-4). In response to metabolic reprogramming, HCC cells express high levels of HK2, which in turn promotes lactate production and glucose consumption in HCC cells [11]. Several mechanisms have been implicated in HK2-driven metabolic reprogramming in HCC: (i) HK2 expression is regulated by multiple oncogenic signaling pathways and transcription factors, including PI3K/AKT/mTOR, AMPK, STAT3, c-Myc, p53, and HIF-1α, which collectively promote HCC development and progression by bypassing growth suppression and activating tumor vasoconstrictor properties [12–16]. (ii) HK2 expression is inversely correlated with glucokinase (HK4, also known as GCK), a key regulator of lipid metabolism, very-low-density lipoprotein (VLDL) secretion, and glycogen storage in normal hepatocytes. This inverse relationship accelerates the malignant transformation of hepatocytes into HCC cells [17]. (iii) HK2 overexpression is associated with increased levels of the immune checkpoint protein PD-L1, with hypoxia-induced glycolysis driving the upregulation of both HK2 and PD-L1 in HCC, thereby promoting chemoresistance [18]. Recent years have seen the development of multiple therapeutic strategies targeting HK2 and its upstream regulatory pathways [11, 19]. Beyond HK2, emerging evidence suggests that in liver fibrosis, TGF-β stimulates the palmitoylation of HK1 in hepatic stellate cells (HSC), thereby promoting the secretion of HK1 via large extracellular vesicles in a TSG101-dependent manner. Upon uptake by HCC cells, HK1 accelerates glycolysis and HCC progression [20]. These findings highlight HK1 as a potential therapeutic target in HCC.

Transcriptional dysregulation is a hallmark of HCC, and phosphofructokinase (PFK), the second rate-limiting enzyme in glycolysis, drives aberrant gene expression by interacting with various transcription factors. In HCC, distinct PFK isoforms exhibit unique regulatory mechanisms: (i) PFKM (PFK muscle isoform) overexpression enhances glycolytic activity and serves as a critical driver of HCC tumorigenesis and intrahepatic metastasis. This effect is mediated by the transcription factor ZEB1, which binds to the E2-box-like sequence CACCT(G) in the promoter region of its target genes [21]. (ii) Elevated PFKL (PFK liver isoform) expression is associated with the downregulation of the zinc-finger transcription factor EGR1, thereby promoting HCC cell proliferation, growth, and resistance to sorafenib [22]. (iii) PFKL also interacts with the E3 ubiquitin ligase A20, whose expression is markedly reduced in advanced HCC tissues. This downregulation contributes to increased glycolysis, as well as enhanced proliferative and invasive capabilities of HCC cells [23]. In summary, inactivating the rate-limiting enzymes involved in the p53-PFK pathway, such as Deoxyribonuclease1-like 3 (DNASE1L3), which is considered a promising prognostic biomarker and therapeutic target for HCC, is also an effective measure to impair glycolysis in HCC cells and tissues [24].

Nowadays, glycolytic reprogramming in HCC has been predominantly centred on the M2 isoform of pyruvate kinase (PKM2), a selectively spliced isoform of the PKM gene, and is also the third rate-limiting enzyme in glycolysis. Post-translational modifications of PKM2, such as acetylation and deubiquitination, enhance HCC responsiveness to immune checkpoint inhibition, thereby facilitating HCC proliferation, invasion, and metastasis [25, 26]. Conversely, suppression of PKM2 activity has been shown to induce apoptosis in both in vivo and in vitro models, for example, lncRNA PWRN1 [27]. It has been confirmed in studies that switching PKM splicing from the cancer-associated PKM2 to the PKM1 isoform with enhanced PKM activity diverts the glucose flux from the PKM2 allosteric regulatory molecule serine synthesis and can inhibit HCC growth [28]. This metabolic switch underscores the pivotal role of PKM splicing in modulating HCC metabolism and proliferation. In addition, PKM2, akin to HK2, is tightly regulated by multiple oncogenic signaling pathways, including the AKT/mTOR/c-Myc axis, AMPK, STAT3, and epithelial-mesenchymal transition (EMT). Through these pathways, PKM2 orchestrates diverse processes essential for HCC progression, highlighting its potential as a therapeutic target [14, 29, 30].

However, due to the energy requirements of rapid cell proliferation, despite the availability of oxygen, the increased metabolic demands of rapidly proliferating HCC cells drive pyruvate decarboxylation catalyzed by pyruvate dehydrogenase (PDH), leading to the excessive production of lactate. Lactic acid has become the main source of nutrition for HCC because it is not only a carbon source for synthesising membrane lipids but also drives the occurrence and development of HCC through epigenetic modifications. Notably, lactylation, a recently identified epigenetic mark arising from lactate accumulation, modulates gene promoter activity associated with N-myc downstream-regulated gene 1 (NDRG1), thereby conferring senescence resistance in HCC cells [31]. Emerging evidence underscores the pivotal role of the PDH complex in driving HCC progression. PDHβ (PDHB), as the E1β subunit of the PDH complex, together with the E1α subunit, facilitates the pyruvate decarboxylation reaction, and its overexpression mediates metabolic reprogramming by binding to the promoter regions of SLC2A1, GPI, and PKM2, promoting the transcription of glycolysis-related genes. This results in the upregulation of glycolytic gene expression, driving HCC cell proliferation, invasion, and sorafenib resistance both in vitro and in vivo [32]. In this context, Isoacteoside, a selective inhibitor of PDHB, has demonstrated synergistic antitumor effects when combined with sorafenib, leading to a significant reduction in tumor volume in HCC mouse models. However, the precise molecular mechanisms underlying its antitumor activity in HCC remain to be fully elucidated [32]. Furthermore, the E2 subunit of the PDH complex, dihydrolipoamide S-acetyltransferase (DLAT), is primarily responsible for converting pyruvate to acetyl-CoA in cooperation with the E1 and E3 subunits. Through its role in histone acetylation, DLAT enhances the expression of glucose transporter 1 (GLUT1), thereby activating EMT and markedly increasing the hepatic metastatic potential of HCC cells [33].

In summary, there is evident functional overlap and upstream–downstream feedback among key enzymes in the glycolytic pathway. PKM2 and HK2 share the AKT/mTOR signaling axis and synergistically regulate the progression of HCC. Alternative splicing of PKM2 (e.g., its conversion to PKM1) and acetylation modifications not only influence the direction of glucose flux but also regulate branched metabolic pathways such as serine biosynthesis. PDHB further extends metabolic regulation to the transcriptional level by directly binding to the promoter regions of SLC2A1, GPI, and PKM2, thereby modulating their transcriptional activity and enhancing the expression of glycolysis-related genes. Meanwhile, lactate, a glycolytic byproduct, is no longer considered merely a metabolic waste but also functions as an epigenetic regulator—through mechanisms such as lactylation—to modulate the expression of genes like NDRG1, playing a significant role in senescence resistance and therapy tolerance in HCC cells.

#### Tricarboxylic acid (TCA) cycle

Pyruvate produced from glycolysis is transported from the cytoplasm to the mitochondria, where it undergoes oxidative decarboxylation to generate acetyl-CoA. Within the mitochondria, acetyl-CoA enters the TCA cycle, serving as the convergent metabolic pathway for the catabolism of glucose, lipids, and proteins, and establishing itself as a central metabolic nexus. Mitochondria are the primary organelles governing nutrient distribution and metabolic pathways in the liver, and the movement of metabolites across the mitochondrial membrane may play a key role in HCC growth [34]. One prominent example of this involves citrate, which exits the mitochondria via the mitochondrial citrate carrier (CIC) and enters the cytoplasm, where it fuels de novo fatty acid synthesis through the citrate-pyruvate shuttle, thereby ensuring a continuous supply of lipids essential for HCC cell proliferation [35]. Within fatty acid metabolism, the intermediate acetyl-CoA is further converted into cholesterol by 3-hydroxy-3-methylglutaryl-CoA (HMG-CoA) synthase and HMG-CoA reductase, enabling adaptive responses to oxidative stress. Then, α-ketoglutarate (α-KG) undergoes a two-step conversion to succinate, which crosses the inner and outer mitochondrial membranes and accumulates in the cytoplasm. In this context, succinic inhibits the α-ketoglutarate-dependent oxygenase, which hydroxylates HIF-1α and subsequently causes proteasomal degradation. Succinic accumulates in large quantities in HCC cells, thereby stabilising and activating HIF-1α, and may be a direct signal messenger linking TCA cycle reprogramming and the development of HCC [35].

The metabolic reprogramming of the TCA cycle is, in part, driven by altered gene expression of proteins involved in this pathway, including mutations and deletions of tumor suppressor genes, which represent hallmark events in HCC pathogenesis. Specifically, HCC cells harboring tumor suppressor gene deficiencies suppress the transcription of glycolysis-related gene PKM, redirecting glucose metabolism from aerobic glycolysis toward a TCA cycle and oxidative phosphorylation (OXPHOS)-dependent state [36]. Copper, a critical enzymatic cofactor for mammals to maintain normal cell activity, has recently been identified as a key modulator of TCA cycle activity. Through its carrier Elesclomol, copper binds directly to lipoacylated TCA cycle components, inducing lipoylprotein aggregation and the loss of iron-sulfur (Fe-S) cluster proteins and subsequent proteotoxic stress, ultimately triggering HCC cell death. Consequently, Fe-S cluster biogenesis factors have been recognized as novel prognostic biomarkers, closely associated with immune infiltration patterns in HCC. This finding underscores the therapeutic potential of copper-based treatments, offering a targeted strategy for tumor suppressor gene-deficient HCC subtypes [36–38].

Moreover, oncogene-driven metabolic reprogramming can also modulate TCA cycle function and mitochondrial homeostasis. The oncogene Gankyrin enhances the transcription of TIGAR through nuclear factor erythroid 2-related factor 2 (Nrf2), TIGAR then regulates the transcription of Nrf2 and Gankyrin through feedback. This regulatory network accelerates the metabolic shift from glycolysis to the pentose phosphate pathway (PPP) and the TCA cycle, promoting the production of NADPH, pentose phosphates, and ATP, which collectively foster HCC development [39].

In summary, the TCA cycle in HCC not only serves as a source of energy production but also functions as a mediator of metabolic–transcriptional crosstalk through metabolites such as citrate and succinate, contributing to immune regulation and oxidative stress responses. Loss of tumor suppressor genes can force HCC cells to become metabolically dependent on the TCA cycle and oxidative phosphorylation. Meanwhile, the oncogene Gankyrin accelerates the metabolic flux from glycolysis toward the pentose phosphate pathway and the TCA cycle by modulating the Nrf2–TIGAR feedback loop. This positive feedback also suggests that oncogene-driven enhancement of TCA function may establish a sustained metabolic activation mechanism in HCC.

#### Pentose phosphate pathway (PPP)

The PPP originates from glucose-6-phosphate, an intermediate of glycolysis, and proceeds through oxidation and group transfer to produce 6-phosphogluconate and glyceraldehyde-3-phosphate, eventually reconnecting with glycolysis. Despite not generating ATP, the PPP provides ribose phosphate and NADPH, which are essential for the energy metabolism and biosynthetic processes of HCC cells. Key enzymes in the PPP include glucose-6-phosphate dehydrogenase (G6PD) in the oxidative branch and transketolase (TKT) and transaldolase (TAL) in the non-oxidative branch [40, 41]. Notably, the expression of these enzymes is significantly elevated in HCC tissues and is positively correlated with increased HCC invasiveness and poor prognosis [42]. G6PD has been shown to promote EMT through activation of the STAT3 signaling pathway, enhancing the migratory and invasive capabilities of HCC cells. This suggests that hyperactivation of the PPP may serve as a marker for HCC metastasis [43]. Moreover, aldolase B (ALDOB) and phosphatase and tensin homolog (PTEN) can directly bind to and inhibit G6PD, thereby suppressing the PPP and preventing HCC development [44, 45].

Oxidative stress plays a pivotal role in the oxidation of thiol (-SH)-containing proteins and enzymes, especially damage by the action of reactive oxygen species such as peroxides [46]. TKT binds to the transcriptional repressor BACH1 to mitigate oxidative stress by reducing glucose flux through glycolysis and enhancing glutathione synthesis [47]. Knockdown of TKT results in ROS accumulation and cell cycle arrest, concurrently elevating ribose-5-phosphate and nucleotide levels, thereby protecting the liver from DNA damage and sensitizing HCC cells to sorafenib treatment [47, 48]. In addition to such metabolic roles, TKT contributes to HCC progression via a non-metabolic mechanism involving nuclear translocation and the EGFR signaling pathway [42]. Through interaction with signal transducer and activator of transcription 1 (STAT1), TKT relocates to the nucleus, where it promotes the binding of histone deacetylase 3 (HDAC3) to the farnesoid X receptor (FXR) promoter, thereby suppressing FXR activity and disrupting bile acid homeostasis. This disruption lowers primary and secondary bile acid levels, providing new avenues for HCC therapy [48].

The regulation of oxidative stress by TAL through the PPP is crucial for modulating the transition from liver cirrhosis to HCC [40]. This process is strictly dependent on polyols produced by NADPH depletion and aldose reductase (AR), which prevents carbon capture in the PPP and developmental retardation of TAL defects to safeguard amino acid biosynthesis and the availability of substrates for the mitochondrial TCA cycle [49]. Conversely, inactivation of the TAL-AR axis will trigger metabolic stress [50]. In TAL deficiency, this axis also regulates genes associated with the pro-inflammatory mTOR pathway, leading to upregulation of genes involved in glucose uptake, glycolysis, the PPP, and de novo lipid synthesis [50, 51]. Clinically, this dysregulation manifests as hepatomegaly and significantly elevated serum alpha-fetoprotein (AFP) levels in HCC patients [52]. Notably, N-acetylcysteine has been demonstrated to prevent HCC development in TAL-deficient mouse models [53].

In summary, the role of the PPP in HCC extends far beyond supplying precursors and reducing power for biosynthetic reactions. Its various branches participate in oxidative stress responses, bile acid metabolism, epigenetic regulation, and the activation of inflammatory signaling. Key nodes within the PPP—G6PD, TKT, and TAL—not only bridge glycolysis and the TCA cycle but also interact with non-metabolic pathways such as the STAT pathway, mTOR, HDAC3, and FXR, forming a network-centred regulatory system with metabolism, signaling, and stress response as its core axes. This network property positions the PPP as both a central hub in HCC metabolic reprogramming and a strategic convergence point for multiple therapeutic targets.

#### Gluconeogenesis

Gluconeogenesis, the reverse process of glycolysis, converts non-carbohydrate such as lactate, glycerol, and gluconeogenic amino acids into glucose or glycogen. In the hepatic gluconeogenesis pathway, phosphoenolpyruvate carboxykinase (PCK) catalyzes the conversion of oxaloacetate to phosphoenolpyruvate. Mammalian cells express two PCK isoforms: the cytoplasmic (PCPEK-C or PCK1) and the mitochondrial (PCPEK-M or PCK2) [54]. Under glucose deprivation, the nuclear transcription factor Y Subunit Alpha (NFYA) v2 is markedly upregulated, inducing the robust expression of both PCK1 and PCK2. This results in elevated ROS levels and apoptosis in HCC cells, highlighting the predominantly tumor-suppressive role of gluconeogenesis in HCC [55].

In contrast, with equal conditions, PCK1 deficiency leads to the accumulation of oxaloacetate, which fuels UDP-GlcNAc biosynthesis and markedly enhances the global O-GlcNAcylation levels, a key nutrient sensor in primary human HCC cells [56]. This modification enhances HCC cell proliferation. Meanwhile, the O-GlcNAcylation of lysine acetyltransferase 5 (KAT5) drives the epigenetic activation of the EMT transcription factor TWIST1 through histone H4 acetylation, and enhances the expression of matrix metalloproteinases (MMP9 and MMP14) via c-Myc acetylation. These changes collectively promote EMT and facilitate lung metastasis in HCC [57]. The above process enhances the cancer stem cell (CSC) properties of HCC cells, including their self-renewal, multilineage differentiation, and unlimited proliferation capacities [58]. The Hippo signaling pathway plays a central role in regulating CSC characteristics, with YAP/TAZ-TEAD acting as the key oncogenic drivers [59]. Specifically, PCK1 promotes Hippo pathway activation, thereby reducing YAP nuclear translocation and inhibiting HCC cell stemness [58]. Beyond PCK1 deficiency, PCK1 phosphorylation disrupts its interaction with insulin-induced genes (INSIG1 and INSIG2), facilitating the transfer of the sterol regulatory element-binding protein 2 (SREBP2) and its cleavage-activating protein (SCAP) complex to the Golgi apparatus. This translocation enhances the transcriptional activity of downstream lipogenic genes, and the resulting increase in lipogenesis is closely associated with poor HCC prognosis [60].

**Fig. 3:**
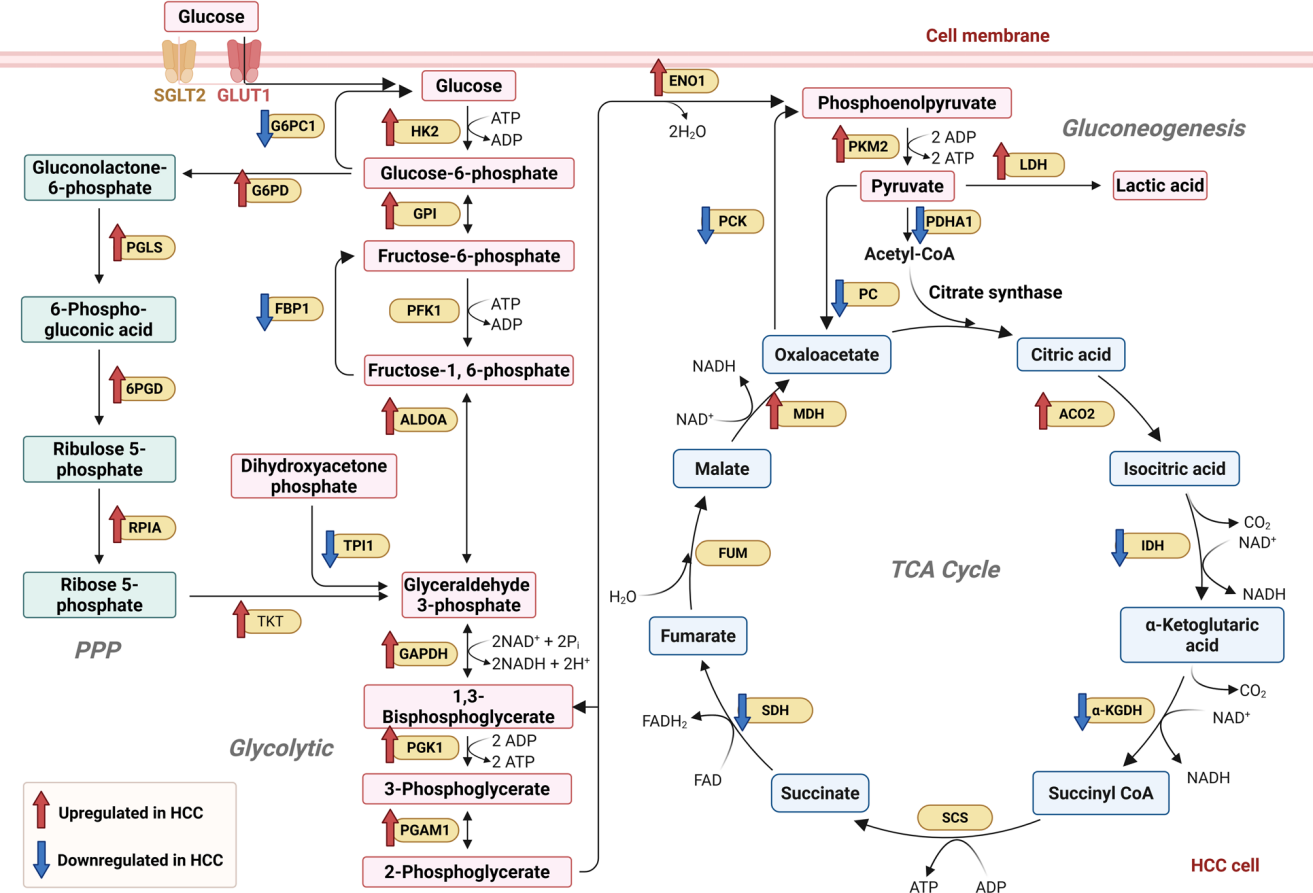
The glucose metabolic reprogramming in HCC. Glucose metabolism contains glycolysis, the TCA cycle, PPP and gluconeogenesis. PGLS, 6-phosphogluconolactonase; 6PGD, 6-phosphogluconate dehydrogenase; RPIA, ribose-5-P isomerase; G6PC1, glucose-6-phosphatase catalytic subunit 1; FBP1, fructose-1,6-bisphosphatase; TPI1, triosephosphate isomerase; TKT, transketolase; GPI, glycosylphosphatidylinositol; ALDOA, aldolase A; GAPDH, glyceraldehyde 3-phosphate dehydrogenase; PGAM1, phosphoglycerate mutase 1; ENO1, enolase 1; PCK, phosphoenolpyruvate carboxykinase; PDHA1, pyruvate dehydrogenase E1 alpha subunit; PC, pyruvate carboxylase; ACO2, aconitase; IDH, isocitrate dehydrogenase; α-KGDH, α-ketoglutarate dehydrogenase; SCS, succinyl CoA synthetase; SDH, succinate dehydrogenase; FUM, fumarase; MDH, malate dehydrogenase. Created with BioRender.com

Subsequently, fructose-1,6-bisphosphatase 1 (FBP1) catalyzes the conversion of fructose-1,6-bisphosphate to fructose-6-phosphate. Following HBV infection, FBP1 expression is significantly upregulated and facilitates the recruitment of the host factor Speckled 110-kDa Protein (SP110) to this site [61]. SP110 further recruits the deacetylase Sirtuin2 (SIRT2), which interacts with and directs the transcriptional activator hepatocyte nuclear factor 4α (HNF4α) to the promoter region. This cascade drives the epigenetic reprogramming of FBP1, leading to reduced transcriptional activity and accelerated HCC progression [61]. Therefore, SP110 has emerged as a potential prognostic marker for viral hepatitis-associated liver cancer [54]. Given its role as a key regulator linking EMT and metabolic reprogramming in HCC, HNF4α is generally downregulated in HCC cells, making it a promising therapeutic target [62]. Overall, these findings suggest that FBP1 appears to be a tumor suppressor in HCC. Figure 3 provides a comprehensive overview of glucose metabolism reprogramming in HCC.

In summary, gluconeogenesis exhibits dual functionality in HCC: its activation contributes to redox balance maintenance and apoptosis induction, suggesting potential antitumor effects; however, when the expression of its key enzymes (PCK1, FBP1) is impaired or their functions are diverted toward lipogenesis, EMT, and stemness maintenance pathways, gluconeogenesis can shift into a pro-tumorigenic mechanism. Its regulatory network spans energy metabolism, epigenetic modification, lipid synthesis, and transcriptional control, and intersects extensively with major oncogenic pathways such as the Hippo pathway, O-GlcNAc signaling, SREBP, and HNF4α. This highlights gluconeogenesis as a metabolically pivotal process with both “protective” and “promoting” potential in HCC, making it a highly networked target for precision intervention.

### Key molecular network of lipid metabolic reprogramming

#### Free fatty acid metabolism

In HCC, fatty acid metabolic reprogramming involves a dynamic balance between de novo fatty acid synthesis and β-oxidation. In terms of anabolic metabolism, key enzymes such as fatty acid synthase (FASN), acetyl-CoA carboxylase 1 (ACC1), elongation of very long chain fatty acids protein 6 (ELOVL6), and acyl-CoA synthetase long-chain family member 4 (ACSL4) are significantly upregulated in HCC, and through c-Myc-mediated upregulation of SREBP1 and its downstream lipogenic enzymes in HCC cells, they constitute the main axis from acetyl-CoA to long-chain fatty acid synthesis [63, 64]. Meanwhile, diacylglycerol lipase α (DAGLA) promotes lipid synthesis and lenvatinib resistance by inhibiting LATS1/YAP phosphorylation in the Hippo pathway, thereby enhancing YAP nuclear translocation [65]. On the other hand, excessive free fatty acids can induce ferroptosis, oxidative stress, and endoplasmic reticulum (ER) stress, serving as sources of metabolic stress signals [66, 67]. FASN induces sorafenib resistance by inhibiting Solute Carrier Family 7 Member 11 (SLC7A11) transcription and reducing glutathione levels [68, 69]; whereas the deletion of SLC27A4 [70] and SLC27A5 [71] can reverse this process, suggesting that free fatty acid metabolism is not only a material supply pathway but also a component of the cellular fate-determining network.

Fatty acid catabolism maintains energy balance in the normal liver primarily through β-oxidation mediated by the CPT, MCAD, LCAD, and other enzymes. As a cholesterol sensor in the endoplasmic reticulum (ER), abnormal expression of SREBP1 can lead to downregulation of MCAD and induction of ER stress, thereby enhancing HCC invasiveness [72, 73]. LCAD deficiency further disrupts signaling homeostasis mediated by PTEN and tensin proteins, promoting HCC progression [74]. At the transcriptional level, peroxisome proliferator-activated receptors (PPARs) γ coactivator-1α (PGC-1α) are key transcriptional regulators of fatty acid oxidation. The three PPAR isoforms (PPARα/γ/δ) and the coactivator PGC-1α form an upstream regulatory axis for fatty acid oxidation, interacting with signaling pathways such as p38 [75], SIRT1 [76], Akt/mTOR [75], and AMPK [77] to create a multilayered regulatory network. This axis directly activates the expression of fatty acyl enzymes, including CPT1, acyl-CoA oxidase (ACOX), LCAD, and MCAD, modulating fatty acid oxidation in mitochondria and peroxisomes. Notably, the physical interaction between ACC and CPT1A coordinates synthesis and oxidation processes, buffering oxidative stress under nutrient-rich conditions and maintaining metabolic homeostasis [78]. However, enhanced β-oxidation may also generate excessive H_2_O_2_, leading to mitochondrial structural damage and functional suppression. The AMPK–SIRT1 pathway, acting as a lipid oxidative stress-sensing module, protects mitochondria by regulating the SIRT1/PGC-1α–PPARα axis. Pharmacological interventions, such as L-carnitine, can activate AMPK to reduce steatosis and prevent HCC development [79]. Finally, the fatty acid oxidation product acetyl-CoA can enter the TCA cycle or generate KB, further linking lipid metabolism with central carbon metabolism and enhancing the energy adaptability of HCC cells.

In summary, fatty acid metabolism in HCC is not limited to a linear pathway of synthesis or degradation but instead forms a complex metabolic network under the regulation of multiple signaling axes such as FASN/SREBP1–YAP, PPARs–AMPK, and CPT1A/ACC. This network possesses the capacity for stress sensing, self-regulation, and therapeutic resistance. Centred on the coordinated regulation of FA synthesis and β-oxidation, it extends in multiple directions toward ferroptosis, endoplasmic reticulum stress, redox balance, ketone body production, and the TCA cycle, serving as a critical hub that links metabolic adaptation, treatment resistance, and microenvironmental response in HCC.

#### Cholesterol and bile acid metabolism

The cholesterol synthesis pathway begins with acetyl-CoA, which undergoes a cascade of enzymatic reactions to generate mevalonic acid (MVA)—a pivotal intermediate in cholesterol biosynthesis, regulated by the rate-limiting enzyme 3-hydroxy-3-methylglutaryl-CoA reductase (HMGCR) [80]. A recent study reported that HCC still develops in FASN-knockout mice due to FASN deficiency-driven SREBP2 activation and subsequent HMGCR upregulation [81]. This results in the overexpression of cholesterol biosynthesis-related genes, elevated cholesterol ester levels, and reduced triglyceride levels in HCC-bearing mice. Following this, MVA is converted into squalene through multiple enzymatic steps. Cytochrome P450 family 51 subfamily A member 1 (CYP51A1), a key P450 enzyme, catalyzes the cyclization of squalene into 7-dehydrocholesterol in the later stages of cholesterol synthesis. Recent findings reveal that Yin Yang 2 (YY2), a C2H2-zinc finger transcription factor, downregulates the transcriptional activity of CYP51A1, thereby limiting cholesterol production. This mechanism alters HCC cell membrane fluidity and disrupts signaling pathways associated with cell cycle, apoptosis, and metabolism, ultimately inhibiting HCC proliferation [82].

Finally, 7-dehydrocholesterol is converted to cholesterol by a reduction reaction, which further promotes the expansion of the CSCs population [83]. Interestingly, a high-fat, high-cholesterol diet (HFHC) drives the sequential progression from fatty liver and steatohepatitis to fibrosis and NAFLD-associated HCC (NAFLD-HCC). A high-fat, low-cholesterol diet only induces hepatic steatosis in mice [84]. This progression is linked to gut microbiota dysbiosis, with microbial composition evolving through NAFLD-HCC stages: Mucispirillum, Desulfovibrio, Anaerotruncus, and Desulfovibrionaceae increase sequentially, while Bifidobacterium and Bacteroides are depleted in HFHC-fed mice [84]. Mechanistically, dietary cholesterol-induced bile acid synthesis and downregulation of the tryptophan metabolite indole-3-propionic acid compromise gut barrier function, exacerbating hepatic triglyceride accumulation and driving NAFLD-HCC progression [84]. Remarkably, the probiotics Bifidobacterium pseudolongum and Lactobacillus acidophilus secrete acetate and valeric acid, respectively, which suppress oncogenic pathways and restore gut barrier integrity, thereby preventing NAFLD-HCC [85, 86]. These findings highlight the importance of maintaining gut barrier function and cholesterol–bile acid homeostasis in reducing HCC aggressiveness, with emerging evidence supporting the roles of transmembrane protein 147 [87], sterol O-acyltransferase 1 [88], and steroidogenic acute regulatory protein 1 [89].

Despite these insights, therapeutic approaches targeting cholesterol metabolism have yet to achieve substantial clinical success, and the long-term prognosis of HCC remains poor. To improve this situation, recent studies propose targeting intrinsic tumor-promoting pathways directly in HCC cells [90, 91]. As mentioned earlier, the transcription coactivators YAP and TAZ and their upstream and downstream mediators are often activated as an intrinsic way to target HCC cells. During cholesterol metabolic reprogramming, MVA indirectly activates YAP1, promoting its nuclear translocation and interaction with nuclear TEAD factors [90]; simultaneously, the MVA–squalene–cholesterol biosynthetic pathway regulates TAZ expression. The CSN6–HMGCS1–YAP1 axis, cholesterol–TAZ–TEAD2 pathway, and TAZ target genes ANLN and KIF23 collectively drive HCC cell proliferation [90, 91]. These pathways offer promising therapeutic targets, exemplified by the small molecule CV-4-26 [92].

In summary, the cholesterol synthesis and bile acid production pathways in HCC are regulated by multiple signaling routes, involving key nodes such as SREBP2, YY2, CYP51A1, HMGCS1, and YAP/TAZ. Through feedback regulation, metabolite sensing, and microbiota modulation, these molecules collectively construct a highly integrated, multilayered network encompassing “cholesterol synthesis–tumor signaling–immune microenvironment–gut barrier.” On this basis, strategies targeting HMGCR, TAZ, YAP, and their downstream genes may offer new breakthroughs for HCC therapy.

#### Sphingolipid metabolism

Sphingolipid metabolism in HCC constitutes a multilayered and multi-pathway metabolic network. Its key regulatory enzymes include sphingosine kinase 1 (SphK1) and sphingosine kinase 2 (SphK2), which catalyze the conversion of sphingosine to sphingosine-1-phosphate (S1P) and regulate the metabolism of ceramides and sphingomyelins. Several studies have shown that the SphK1/S1P axis is activated in HCC cell lines [93]. SphK1 induces EMT in HCC cells by stimulating autophagy, and as an upstream kinase, it activates the PI3K/AKT, NF-κB, and MAPK pathways via S1P receptors, thereby enhancing HCC cell proliferation and migration. It is associated with poor prognosis and oxaliplatin resistance in HCC patients and thus represents a potential independent prognostic biomarker for HCC therapy [93–95]. In contrast, SphK2 deficiency is linked to sex-specific tumor susceptibility, characterized by significantly reduced p62 levels in male mice, which weakens high-fat diet–induced abnormal hepatocyte proliferation [96]. This sex-specific response suggests that the sphingolipid metabolic network is regulated not only by enzyme activity but also by hormonal or genetic background. As a dual regulator of autophagy and metabolism, p62 cooperates with IMP2 to activate Wnt/β-catenin signaling, further promoting EMT and invasive progression in HCC [97]. Meanwhile, membrane lipidomic analysis has revealed that Wnt/β-catenin activation is accompanied by a significant reduction in ceramide and diacylglycerol levels, indicating the existence of a bidirectional feedback mechanism between sphingolipid components and signaling pathways [98].

Moreover, compared with normal liver tissue, the levels of other sphingolipids upstream of S1P in the metabolic cascade—such as sphingomyelin (SM), glucosylceramide, and ceramide (Cer)—are also markedly elevated in HCC tissues [99]. Neutral sphingomyelinase 1 (NSMase1), an enzyme that converts SM to Cer, is significantly downregulated in HCC, leading to a decreased SM/Cer ratio, which is strongly associated with poor prognosis in HCC patients [95]. SphK2 deficiency results in pronounced sphingolipid reprogramming, with substantial increases in upstream intermediates including dihydroceramide, Cer, dihydrosphingosine, and SM, a process related to the downregulation of ceramide transfer protein (CERT) [100]. In summary, SM, Cer, and the SphK1/SphK2-mediated sphingolipid metabolic axis together form a complex network of coordinated regulation among signaling molecules, membrane lipid architecture, and metabolic enzymes.

**Fig. 4:**
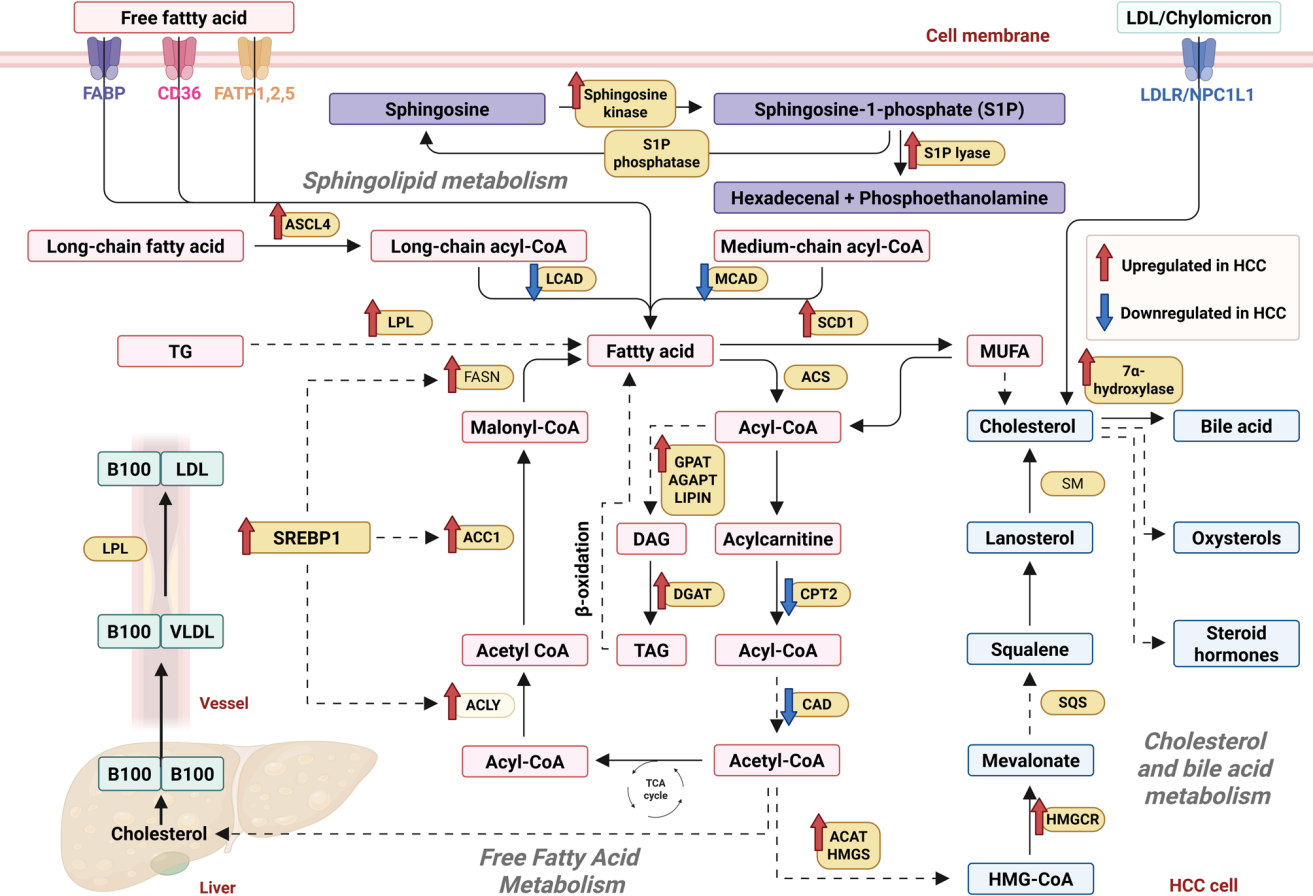
The lipid metabolic reprogramming in HCC. Lipid metabolism contains free fatty acid metabolism, cholesterol and bile acid metabolism, and sphingolipid metabolism. ASCL4, acyl-CoA ligase 4; LPL, lipoprotein lipase; LCAD, long chain acyl-CoA dehydrogenase; MCAD, medium chain acyl-CoA dehydrogenase; ACC1, acetyl-CoA carboxylase 1; ACLY, ATP citrate lyase; ACS, acyl-CoA synthetases; GPAT, glycerol-3-phosphate-1-acyltransferase; AGPAT, 1-acylglycerol-3-phosphate-O-acyltransferase; DGAT, diacylglycerol acyltransferase; CPT2, carnitine palmitoyltransferase 2; CAD, carnitine acyltransferase I; ACAT, acyl-coenzyme A: cholesterol transferase; HMGS, 3-hydroxy-3-methylglutaryl-CoA synthase; SM, squalene monooxygenase; SQS, squalene synthase; HMGCR, HMG-CoA reductase. Created with BioRender.com

In summary, sphingolipid metabolism in HCC is not merely an isolated lipid alteration process but rather a complex system involving multiple lipid mediators, signaling pathways, and multilayered regulatory factors. Core network nodes—such as SphK1/SphK2, p62, S1P, ceramide, and their receptors—interconnect multiple pathways, including PI3K/AKT, Wnt/β-catenin, p38, and NF-κB, collaboratively regulating cellular metabolism, signal responses, and the immune microenvironment. Targeting key hubs of this network, particularly SphKs and their downstream lipid receptors, may offer significant breakthroughs for early HCC diagnosis, prognostic evaluation, and personalized intervention. Figure 4 provides an overview of the reprogrammed lipid metabolic pathways in HCC.

### Key molecular network of amino-acid metabolic reprogramming

#### Glutamine metabolism

Glutamine plays a central role in energy production, cell survival, the maintenance of signaling pathways associated with HCC proliferation, and the provision of NADPH and glutathione (GSH) for redox homeostasis [101]. In mammals, ammonia and glutamate are converted into glutamine by the enzyme glutamine synthetase (GS), which is often overexpressed in HCC and closely associated with β-catenin activation [102]. Glutamine breakdown begins with the mitochondrial enzyme glutaminase (GLS), which catalyzes the conversion of glutamine to glutamate, followed by the transformation of glutamate into α-ketoglutarate. There are two isoforms of GLS: GLS1, which functions as an oncogene, and GLS2, which acts as a tumor suppressor. Further research confirmed that GLS2—rather than GLS1—promotes α-ketoglutarate production and induces ferroptosis by enhancing lipid ROS formation, thereby inhibiting HCC development [103]. The downregulation of GLS2 has been linked to increased HCC invasiveness and poor patient prognosis [103].

As glutamine is primarily cytoplasmic, its catabolism requires specific transporters to cross the outer mitochondrial membrane [104]. Confirmed glutamine transporters include the SLC1A5 variant [105] and ASCT2 [106]. Under hypoxic conditions, the expression of these transporters is upregulated, facilitating enhanced glutamine catabolism and redox regulation in HCC cells [105, 106]. Consequently, human HCC cells exhibit a strong dependence on exogenous glutamine, often described as “glutamine addiction” [107]. However, HCC cells may not decrease as expected when deprived of glutamine due to nutritional deficiencies. This is because branched-chain amino acid catabolism can sustain HCC progression by bypassing glutamine dependence via the O-Linked N-Acetylglucosamine-Protein Phosphatase [108]. Additionally, HCC cells adapt to glutamine deprivation by shifting their metabolic balance, reducing fatty acid synthesis, and increasing fatty acid oxidation [109]. Notably, dual inhibition of glutamine metabolism and these compensatory pathways significantly suppress tumor growth in HCC mouse models, underscoring the potential of combination therapies in HCC treatment [108, 109].

Following glutamine catabolism, α-ketoglutarate undergoes further oxidation to succinate, entering the TCA cycle for ATP production. Alternatively, it can engage in the reductive arm of the TCA cycle, promoting lipid synthesis [110]. Glutamate-oxaloacetate transaminase (GOT) catalyzes the bidirectional conversion between glutamate and α-ketoglutarate and is highly specific to liver tissue [111]. There are two GOT isoforms: GOT1, localized in the cytoplasm, and GOT2, found in the mitochondria. In the liver, GOT2 is predominant and catalyzes the conversion of glutamate and oxaloacetate to α-ketoglutarate and aspartate, while GOT1 facilitates the reverse reaction [112, 113]. Recent studies have shown that GOT2 is significantly downregulated in HCC, driving metabolic reprogramming that enhances glutamine catabolism and GSH synthesis. The resulting antioxidant capacity reduces intracellular ROS levels and boosts ATP production, thereby promoting HCC proliferation, hematogenous spread, and intrahepatic metastasis—processes associated with late-stage disease and poor prognosis [113]. These findings suggest that GOT2 could serve as a potential biomarker for the efficacy of glutaminase inhibitors in HCC therapy [113].

In summary, glutamine metabolic reprogramming in HCC is not only characterized by energy acquisition and redox balance regulation but also forms a highly integrated metabolism–signaling regulatory network through GLS, GOT, transporters, and their associated pathways (such as Wnt/β-catenin, HIF, O-GlcNAc, and lipid signaling). This network possesses both metabolic flexibility and adaptive feedback capacity, linking carbon and nitrogen metabolism, fatty acid metabolism, and ferroptosis regulation. It represents a classic hub of metabolic remodeling and therapeutic resistance in HCC.

#### Cysteine metabolism

Cysteine serves as the rate-limiting precursor for GSH biosynthesis. α-ketoglutarate produced by the above metabolic pathway enters the TCA cycle to produce downstream metabolites, including succinate, fumarate, and malate, which can induce mitochondrial oxidative stress, thereby promoting lipid ROS accumulation and ferroptosis in HCC under conditions of cysteine deprivation [114, 115]. Thus, the suboptimal antitumor efficacy of sorafenib may be attributed to the presence of alternative cysteine acquisition pathways in HCC, among which macropinocytosis has been identified as a key mechanism [116]. As a distinctive endocytic process in mammalian cells, macropinocytosis shares several organelle features with the phagosome-lysosome system; however, macropinocytosis is uniquely induced by the activation of growth factor signaling pathways rather than direct receptor regulation [117]. Sorafenib stimulates macropinocytosis by targeting mitochondrial biogenesis and activating the Phosphoinositide 3-Kinase - Ras-Related C3 Botulinum Toxin Substrate 1 - p21-Activated Kinase 1 (PI3K-RAC1-PAK1) signaling axis, which facilitates the degradation of extracellular proteins to compensate for sorafenib-induced cysteine deprivation, thereby preventing ferroptosis in HCC cells [116]. Targeting macropinocytosis has emerged as a promising strategy to enhance the therapeutic sensitivity of advanced HCC, underscoring the potential of novel combination therapies.

Cysteine and cystine are interconvertible under specific conditions, and modulating their levels in HCC tissues may provide valuable insights into identifying therapeutic targets. Firstly, depleting cystine levels or inhibiting the cystine transporter SLC7A11 reduces intracellular cysteine availability, thereby impairing GSH biosynthesis and promoting ROS accumulation in HCC cells [118]. Ubiquitin-specific peptidase 8 (USP8) has been demonstrated to stabilize O-linked N-acetylglucosamine transferase (OGT) through its deubiquitination activity, thereby enhancing the O-GlcNAcylation of SLC7A11 (a post-translational modification) and facilitating cystine uptake in HCC cells—a process closely associated with the high proliferative capacity and clonogenicity of HCC, making USP8 a potential therapeutic target [119]. Secondly, inhibiting selenocysteine biosynthesis disrupts GSH metabolism by inactivating glutathione peroxidase 4 (GPX4) [120]. Phosphoseryl-tRNA kinase (PSTK) 4, a critical intermediate in selenocysteine synthesis, has been identified as a key modulator of ferroptosis sensitivity in HCC cells, with PSTK4 knockout significantly enhancing the efficacy of ferroptosis-targeted chemotherapy [120]. Finally, the γ-glutamylcysteine synthetase heavy chain (γ-GCSh), the rate-limiting enzyme in GSH biosynthesis, has been implicated in mediating resistance to chemo- and radiotherapy. Comparative analyses of the γ-GCSh-overexpressing GCS30 subline and its parental line revealed that the former exhibited significantly enhanced resistance to apoptosis following exposure to 10–20 Gy doses of radiotherapy or treatment with chemotherapeutic agents such as arsenic trioxide (ATO), cisplatin, or doxorubicin [121]. These findings suggest that targeting γ-GCSh to inhibit cysteine synthesis represents a promising approach to overcoming therapy resistance in HCC cells [121].

In summary, cysteine metabolism in HCC, through key regulators such as SLC7A11, USP8–OGT, PSTK–GPX4, and γ-GCSh, establishes a complex metabolism–signaling interaction network that governs ferroptosis, antioxidant defense, and therapeutic resistance. This network not only maintains cysteine and GSH supply through multiple pathways but also interacts extensively with PI3K, O-GlcNAc signaling, and radiotherapy response mechanisms, exhibiting highly integrated features of metabolic adaptability, treatment evasion, and signaling feedback. Targeting multiple regulatory hubs within this network may overcome the limitations of single-point interventions and offer new avenues for precision therapy in HCC.

#### Branched chain amino acid (BCAA) metabolism

As the second most abundant nitrogen source in cells following glutamine, the catabolism of BCAA, including isoleucine, leucine, and valine, is upregulated in HCC cells under glutamine-deprived conditions. This metabolic shift channels BCAA-derived carbon and nitrogen into nucleotide biosynthesis pathways, thereby accelerating cell cycle progression and promoting HCC cell survival [108]. The degradation of BCAA is initiated by the branched-chain aminotransferases BCAT1 and BCAT2, which are key metabolic proteins responsible for glutamate production [122]. Notably, BCAT1 has been shown to enhance HCC cell resistance to cisplatin by inducing autophagy via the mTOR pathway [123] and to facilitate HCC invasion and metastasis through activation of the Akt signaling pathway and EMT [124]. Conversely, BCAT2, regulated by the AMPK-SREBP1 signaling axis, functions as a negative regulator of ferroptosis by maintaining intracellular glutamate homeostasis, thereby protecting the liver from both endogenous and exogenous ferroptosis triggers and representing a potential therapeutic target for overcoming sorafenib resistance [125].

Following transamination, the rate-limiting enzyme BCKDH catalyzes the conversion of BCAA into branched-chain α-keto acids (BCKAs), which subsequently fuel the TCA cycle through the production of succinyl-CoA, acetoacetate, and propionyl-CoA. The mitochondrial pyruvate carrier (MPC), a heterodimeric complex embedded in the mitochondrial inner membrane, governs the metabolic interplay between mitochondrial pyruvate and BCAA catabolism. Inhibition of MPC has been demonstrated to enhance BCAA degradation by promoting BCKA oxidation and downregulating BCKDH phosphorylation in vitro, while concurrently activating mTOR signaling in vivo, thereby reinforcing BCAA catabolic flux in HCC cells [126, 127].

Overall, BCAA metabolism, through BCAT1/2, BCKDH, and its upstream regulatory axes (such as mTOR, Akt, AMPK, and SREBP1), forms a multilayered metabolic network that regulates energy supply, signaling pathway activation, antioxidant defense, and therapeutic resistance. This network not only exhibits compensatory interplay with glutamine metabolism but also intersects with the TCA cycle, ferroptosis, pyruvate metabolism, and autophagy, collectively driving the adaptive survival of HCC cells under nutrient stress. Therefore, precisely targeting this metabolic hub may represent a promising strategy to overcome current therapeutic bottlenecks in HCC.

#### Asparagine metabolism

As a downstream metabolic product of glutamine, asparagine plays a pivotal role in HCC growth and metastasis by regulating mitochondrial respiration, cell survival, and proliferation, and the mTOR pathway, directly involved in the growth and metastasis of HCC [128]. Asparagine synthetase (ASNS) catalyzes the conversion of aspartate to asparagine, and its upregulation has been associated with the enhanced growth of drug-resistant tumors [129]. Conversely, asparaginase (ASNase) catalyzes the conversion of asparagine back to aspartate and acts as an inhibitor of ASNS [130]. In HCC cells, studies have identified pituitary tumor-transforming gene 1 (PTTG1) as an oncogene involved in proliferation and metabolism, which is significantly enriched in the asparagine metabolic pathway. PTTG1 enhances HCC proliferation by upregulating ASNS transcription and activating the mTOR pathway [131]. Further investigations have revealed that HCC subgroups with elevated asparagine metabolism are associated with an immunosuppressive microenvironment. ASNS and its core gene GOT2 demonstrate significant predictive value for OS, with their low expression correlating with poor prognosis. Therefore, they represent promising prognostic biomarkers and therapeutic targets for HCC [132, 133]. Regarding combination therapy, future strategies may involve the co-administration of the tyrosine kinase inhibitor lenvatinib and ASNase to induce oxidative stress, offering a novel therapeutic approach for HCC treatment [134].

Glycosyltransferases play a crucial role in controlling the migratory capacity of HCC cells. To elucidate the complex mechanisms underlying the poor prognosis of HCC, we next integrate glycoproteomics for further analysis. N-glycosylation, a co-translational or post-translational modification of nascent polypeptides, is essential for cell communication and signaling, HCC cell adhesion, immune regulation, and angiogenesis [135]. The most common form of N-glycosylation is asparagine-linked glycosylation. AFP, a glycoprotein with a single N-linked glycosylation site at asparagine residues 251, has been widely used in clinical HCC diagnostics [136]. Compared with adjacent normal liver tissues, the transcription and translation levels of asparagine-linked glycosylation 1 homolog (ALG1), an asparagine-linked glycosylation protein homolog, are significantly downregulated in HCC. This reduction inhibits the expression of N-cadherin, thereby preventing cell-cell adhesion, increasing histopathological grading, and accelerating HCC invasion and metastasis [137]. These findings suggest that targeting N-glycosylation inhibition may represent a potential therapeutic strategy for HCC. Cluster of differentiation 147 (CD147), a transmembrane glycoprotein, undergoes N-glycosylation at asparagine residues 152 in the ER, ensuring proper folding and stability. Loss of N-linked glycosylation at this site disrupts the tumor-associated glycan profile of CD147 and accelerates its degradation [138]. Targeting the asparagine 152 on CD147 significantly suppresses the metastasis of in situ HCC cells, representing a promising avenue for HCC therapy [138]. Moreover, tyrosine receptors play a critical role in the oncogenic regulation of N-glycosylation [139]. Mer tyrosine kinase (MerTK) undergoes N-glycosylation at asparagine residues 294 and 454 in HCC cells, stabilizing MerTK to promote oncogenic transformation. Conversely, MerTK depletion or loss effectively inhibits hepatocarcinogenesis [140].

Taken together, asparagine metabolism plays a central role in nutrient regulation, redox homeostasis, and the immune microenvironment in HCC through the ASNS/PTTG1/mTOR axis. Moreover, its downstream metabolic products expand its impact by participating in N-glycosylation modifications, thereby influencing protein stability, intercellular communication, and cell migration. The interplay with pathways involving GOT2, mTOR, N-cadherin, and MerTK suggests that asparagine functions not merely as a metabolic substrate but as a multidimensional network node driving HCC initiation and progression, with significant diagnostic and therapeutic implications.

#### Serine metabolism

Serine is a critical metabolite involved in numerous biosynthetic processes, including amino acid transport, lipid and nucleotide synthesis, methylation reactions, and the one-carbon cycle, which supports antioxidant defense. As such, many cancer cells, including HCC, exhibit a strong dependence on serine for their growth and proliferation [141]. In HCC cells, intracellular serine levels are maintained through both exogenous uptake and de novo synthesis from glucose, with phosphoglycerate dehydrogenase (PHGDH) serving as the first and rate-limiting enzyme in this biosynthetic pathway [142]. Serine metabolic reprogramming is closely associated with post-translational modifications (PTMs), and several PTMs—including arginine methylation, lysine succinylation, Keap1 ubiquitination, O-GlcNAcylation, and GPX4 dephosphorylation—have been implicated in regulating serine metabolism in HCC [143–147]. Specifically, PRMT1 and lysine succinyltransferase enhance PHGDH activity, leading to increased mitochondrial membrane potential and respiration, which promotes serine synthesis, mitigates ROS accumulation, alleviates oxidative stress, and ultimately drives HCC progression [144, 145]. Meanwhile, TRIM25 directly targets Keap1 for ubiquitination and degradation via its E3 ubiquitin ligase activity, activating the Nrf2 signaling pathway and reducing ROS levels, thereby enhancing HCC cell survival under ER stress conditions [143].

Beyond serine synthesis, PHGDH also plays a pivotal role in activating the tumor suppressor gene p53, which is critical for cell survival under serine deprivation [148, 149]. As a p53-binding protein, PHGDH interacts with AXIN and HIPK2 to form a multivalent p53-binding complex when not occupied by 3-phosphoglycerate (3-PGA), promoting apoptosis and suppressing HCC growth [148]. Dysregulation of wild-type p53 is a key driver of HCC development, and it has been demonstrated that PHGDH mutants with constitutive 3-PGA binding inhibit p53 activation, thereby promoting HCC proliferation [148]. Therefore, targeting PRMT1 for degradation or preventing p53 degradation could disrupt these oncogenic processes and mitigate lung metastasis, with ubiquitination playing a crucial role—exemplified by the involvement of E3 ubiquitin ligases such as F-box-only protein 7 (FBXO7) [150] and WD repeat and SOCS box containing protein 2 (WSB2) [151].

Furthermore, serine can enter various amino acid, glucose, and lipid metabolic pathways under specific conditions and enzymatic regulation, thereby influencing HCC progression. For instance: (i) Catalyzed by serine hydroxymethyltransferase (SHMT), serine is converted to glycine, contributing a one-carbon unit to tetrahydrofolate to generate 5,10-methylene tetrahydrofolate (CH_2_-THF) [141]. Meanwhile, SHMT overexpression suppresses excessive ROS production and EMT, significantly reducing the risk of HCC lung metastasis [152]. (ii) Under the action of serine dehydratase, serine is converted to pyruvate, integrating into the glycolytic reprogramming of HCC [153]. (iii) Through the calcium-dependent activity of phosphatidylserine synthases (PSS1 and PSS2), serine forms phosphatidylserine, which is subsequently decarboxylated and methylated to produce phosphatidylcholine, thus contributing to the lipid metabolic reprogramming in HCC [154].

In conclusion, serine metabolism forms a highly integrated metabolic–signaling network through the PHGDH–p53 axis, the PRMT1/Nrf2 regulatory module, and its interactions with one-carbon metabolism, glycolysis, and phospholipid metabolism. This network coordinately regulates the growth, anti-apoptotic capacity, oxidative stress adaptability, and metastatic potential of HCC cells, providing a theoretical foundation for the precise intervention of HCC through multi-target combination therapy.

#### Proline metabolism

As a non-essential amino acid, proline has been widely recognized as both a regulatory factor and a stress marker in tumorigenesis, with its expression levels showing a positive correlation with liver growth rate and a negative correlation with HCC prognosis [155–157]. Proline biosynthesis in human and animal liver cancer is driven by the significant upregulation of its anabolic enzymes, pyrroline-5-carboxylate reductase (PYCR1) and aldehyde dehydrogenase 18 family member A1 (ALDH18A1), alongside the notable downregulation of its sole catabolic enzyme, proline dehydrogenase (PRODH) [158]. Synthesized proline serves two primary functions: contributing to extracellular matrix formation through collagen production [159] and generating hydroxyproline via prolyl-4-hydroxylase, which stabilizes HIF-1α under hypoxic conditions [160]. Moreover, proline itself exhibits ROS-scavenging properties, protecting HCC cells from ROS-induced cell death, which has drawn increasing research attention to the roles of its synthetic and catabolic enzymes in hypoxia-driven HCC progression [161].

Under hypoxia, PYCR1 phosphorylation at tyrosine 135 by insulin-like growth factor receptor 1 (IGFR1) enhances ELK4-mediated transcriptional repression, a mechanism essential for sustaining colorectal cancer cell growth. The NADH oxidation catalyzed by PYCR1 is also crucial for maintaining TCA cycle activity [162], highlighting the need to further explore its role in HCC and other liver cancer types under hypoxic conditions. Similarly, ALDH18A1 stabilizes HIF-1α in hypoxic HCC cells by inhibiting its hydroxylation through the glutamine, proline, and hydroxyproline metabolic axis, thereby supporting the hypoxia-dependent phenotype of HCC [163].

On the catabolic side, the conversion of pyrroline-5-carboxylate (P5C) back to proline by PYCR1 is vital for sustaining PRODH activity. PRODH/POX (proline oxidase) works in concert with P5C reductase to facilitate the proline shuttle between the mitochondria and cytoplasm, maintaining redox balance and metabolic homeostasis. In breast cancer and melanoma, PRODH/POX has been shown to catalyze proline degradation, generating ROS that induce tumor cell apoptosis or autophagy [164, 165]. This mechanism remains underexplored in HCC and warrants further investigation. Notably, targeting PRODH2 has demonstrated the potential to reshape metabolic pathways and gene expression programs, enhancing the in vivo antitumor efficacy of chimeric antigen receptor (CAR-T)-based therapies [166].

In summary, proline metabolism, through the PYCR1–ALDH18A1–HIF-1α axis–mediated biosynthetic regulation and the potential catabolic signaling via PRODH/POX, jointly contributes to the regulation of HCC cell growth, hypoxia adaptation, and ROS clearance. This forms a functional network closely interacting with glutamine metabolism, hypoxic signaling, the TCA cycle, and immune responses, offering new intervention strategies for future combined metabolic and immuno-targeted therapies.

#### Hexosamine biosynthetic pathway (HBP)

The HBP is a branch of glucose metabolism that shares its initial two steps with glycolysis. Catalyzed by glucosamine-6-phosphate isomerase 1 (GNPDA1), HBP can divert into the glycolytic pathway by converting glucosamine into fructose-6-phosphate. This metabolic shift enhances HCC cell proliferation, invasion, and metastasis while inhibiting apoptosis, ultimately leading to poor patient prognosis [167]. A small fraction of glucose is also converted via HBP into UDP-GlcNAc. In HCC cells, OGT utilizes UDP-GlcNAc to glycosylate serine and threonine residues—a modification closely linked to HCC cell growth and survival. Conversely, O-GlcNAcase (OGA) is the sole enzyme responsible for removing this reversible modification [168–170].

In HCC cells, O-GlcNAcylation regulates the expression of translation factors and ribosomal proteins and interacts with other PTMs. The liver, being the central organ for dietary fructose metabolism, converts fructose-derived acetate into UDP-GlcNAc, thereby promoting O-GlcNAcylation in HCC. Notably, an analysis revealed that hyper-O-GlcNAcylation of eukaryotic elongation factor 1A1 significantly enhances HCC cell proliferation [171]. In the meantime, glucose-deprived HCC cells exhibit a 7.8-fold increase in total O-GlcNAcylation compared to those cultured in normal glucose. This surge is attributed to elevated OGT levels and reduced OGA activity, resulting in glycogen synthase inactivation and decreased PCK1 activity [56, 172]. The loss of PCK1 drives two critical oncogenic processes: (i) It enhances O-GlcNAcylation at the T378 autophosphorylation site of CHK2, a cell cycle checkpoint kinase, subsequently activating CHK2-dependent Rb phosphorylation and promoting HCC cell proliferation [56]; (ii) It induces hyper-O-GlcNAcylation of KAT5, inhibiting its ubiquitination and stabilizing the protein. This stabilization activates TWIST1 expression through histone H4 acetylation and upregulates MMP9 and MMP14 via c-Myc acetylation, thereby facilitating EMT [57].

Beyond its interplay with phosphorylation and ubiquitination, O-GlcNAcylation also crosstalks with ADP-ribosylation. In HCC cells, O-GlcNAcylation of poly(ADP-ribose) glycosidase (PARG) at the Ser26 site enhances poly-ADP-ribosylation of DNA damage-binding protein 1 (DDB1), reduces its ubiquitination, and stabilizes DDB1. This stabilization results in the degradation of downstream targets such as c-MYC, Snail, and β-catenin, effectively suppressing HCC progression [173]. Interestingly, both glucose deprivation and hyperglycaemia amplify O-GlcNAcylation. Specifically, the receptor for advanced glycation end products (AGER) signaling pathway, in a ligand- and cell-type-dependent manner, promotes O-GlcNAcylation of the proto-oncogene c-Jun, enhancing its activity and stabilizing it through an AGER/OGT-dependent mechanism, thereby driving liver tumorigenesis [174].

**Fig. 5:**
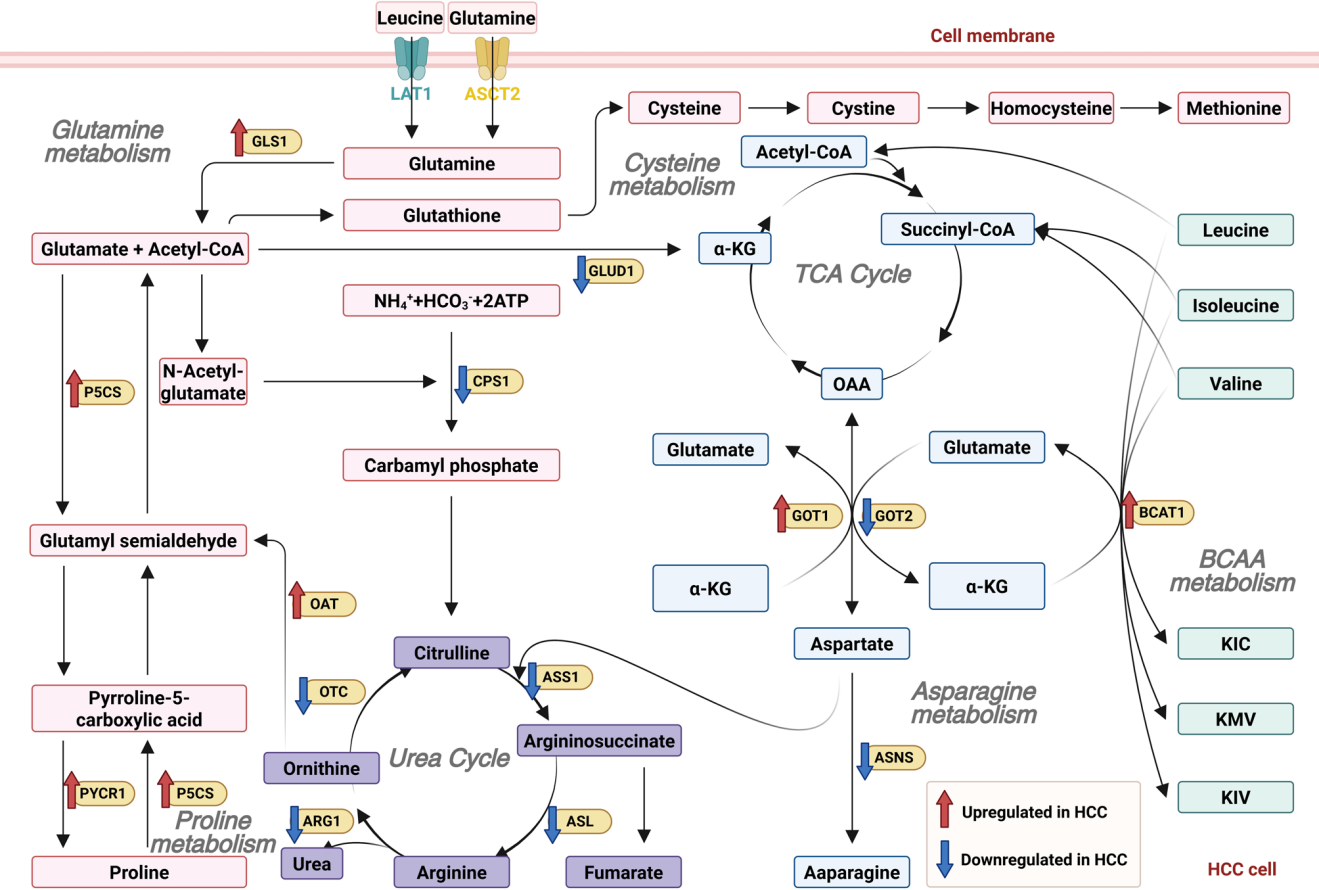
The amino acid metabolic reprogramming in HCC. Amino acid metabolism contains glutamine, cysteine, branched chain amino acids, asparagine metabolism, and more. P5CS, pyrroline-5-carboxylate synthase; PYCR1, pyrroline-5-carpoxylate reductase-1; GLUD1, glutamate dehydrogenase 1; OAT, ornithine aminotransferase; OTC, ornithine transcarbamylase; ARG1, arginase-1; ASS1, argininosuccinate synthetase 1; ASL, argininosuccinate lyase; GOT1/2, glutamate oxaloacetate transaminase 1/2; ASNS, asparagine synthetase; BCAT1, branched-chain amino acid transaminase 1. Created with BioRender.com

In summary, HBP, by linking glycolysis to UDP-GlcNAc production, regulates OGT-mediated O-GlcNAc post-translational modification. In HCC, it extensively intersects with other PTMs such as phosphorylation, ubiquitination, and ADP-ribosylation, forming a highly coupled signaling network that integratively controls cellular metabolic status, genetic regulation, and phenotypic evolution. This provides an important foundation for multi-target combinatorial intervention strategies. Figure 5 summarizes the reprogramming of amino acid metabolism pathways in HCC.

## Crosstalk of HCC metabolic pathways

**Fig. 6:**
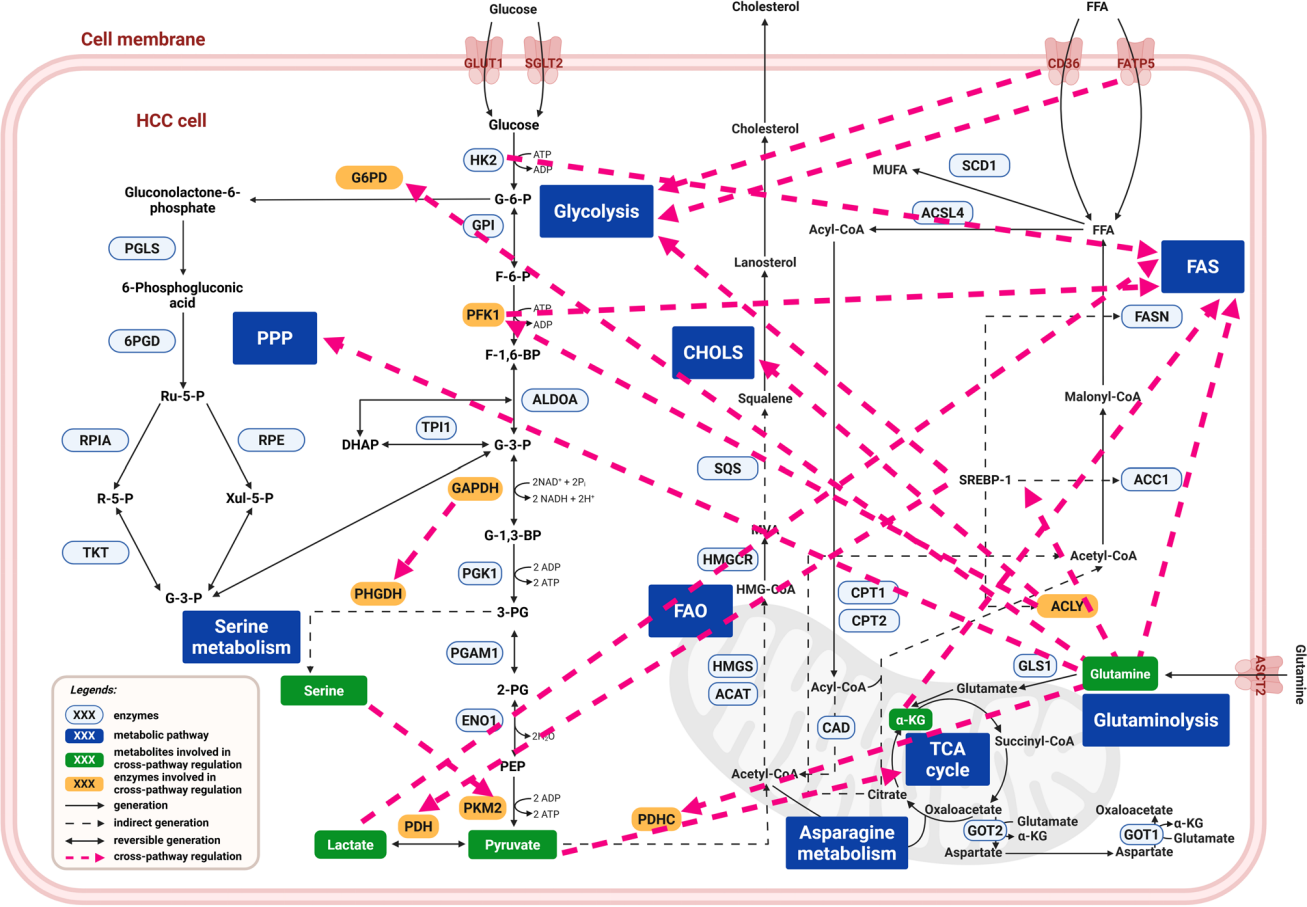
Cross-pathway regulation by enzymes/metabolites between glucose, lipid and amino acid metabolism in HCC. Glycolysis and the TCA cycle are central hubs for glucose utilization in various metabolic pathways of HCC cells, such as glycolysis, the pentose phosphate pathway (PPP), free fatty acid metabolism, cholesterol metabolism, glutamine metabolism, asparagine metabolism, and serine metabolism. Exploring the cross-pathway regulatory mechanisms mediated by enzymes and metabolites helps to elucidate the mechanisms supporting HCC progression and provides potential targets for achieving precision diagnosis and therapy. Created with BioRender.com

Metabolic reprogramming in HCC involves not just the activation or inhibition of individual metabolic pathways but rather intricate crosstalk and coordinated regulation among multiple metabolic networks. This metabolic interplay is particularly prominent among glucose, lipid, and amino acid metabolism. Based on current research, the crosstalk between these three metabolic processes can be categorized into four major dimensions: the dynamic reprogramming of metabolic networks, the interconversion of metabolic intermediates, the cross-regulation of key metabolic regulators, and the maintenance of energy homeostasis. Together, these mechanisms equip HCC cells with remarkable metabolic plasticity and adaptability, enabling them to survive and proliferate even under the adverse conditions of the tumor microenvironment. Figure 6 illustrates the cross-pathway regulation by enzymes/metabolites between glucose, lipid, and amino acid metabolism in HCC.

### Crosstalk between glucose and lipid metabolism

In the metabolic reprogramming of HCC, glucose metabolism plays a critical role in modulating lipid metabolism, with the underlying molecular mechanisms involving three key aspects: (i) Stromal interaction molecule 1 (STIM1), a Ca^2+^ sensor in the ER, triggers Ca^2+^ influx from calcium stores, stabilizing HIF-1α through the activation of Ca^2+^/calmodulin-dependent protein kinase II (CaMKII). In HCC cells, enhanced glucose metabolism is accompanied by activation of the STIM1–CaMKII–HIF-1α axis, which not only increases glycolytic flux but also promotes lactate accumulation. The latter, by acidifying the microenvironment and acting as a metabolic signal, enhances lipid synthesis and facilitates HCC invasion [175]. (ii) Carbohydrate-responsive element-binding protein (ChREBP), a key transcription factor mediating hepatic glucose signaling, Glucose metabolism enhances lipid synthesis pathway activity by inducing ChREBP to upregulate key lipid metabolic enzymes such as ACLY and FASN. Meanwhile, GCK activity is essential for maintaining the cooperative induction of lipogenic genes by ChREBP and SREBP-1c; its deficiency significantly impairs the coupling efficiency between glucose and lipid metabolism [176, 177]. (iii) Oroxyloside (OAG), a novel dual agonist of PPARγ/α, suppresses aerobic glycolysis while promoting fatty acid oxidation, primarily by supplying acetyl-CoA to fuel the TCA cycle and oxidative phosphorylation [178]. This OAG-induced metabolic shift markedly elevates ROS levels, leading to rapid RB dephosphorylation, G1 cell cycle arrest, and growth suppression in HCC cells, ultimately contributing to sorafenib resistance [178].

On the other hand, lipid metabolism reciprocally influences glucose metabolism through two primary mechanisms: (i) CD36, a fatty acid transporter highly expressed in the liver, muscle, and adipose tissues, activates the mTOR pathway via the Src/PI3K/AKT signaling cascade, significantly enhancing lactate production in HCC cell lines [179]. Inhibition of mTOR or CD36 depletion reduces the high-affinity uptake of long-chain fatty acids, impairs glucose metabolism, and disrupts the activation of PPAR and AMPK [180]. (ii) Aldo-keto reductase family 1 member C3 (AKR1C3)-dependent lipid droplet formation mitigates mitochondrial lipotoxicity and suppresses fatty acid oxidation, driving a metabolic shift toward glycolysis in HCC cells. This metabolic adaptation protects HCC cells from acylcarnitine-induced mitochondrial dysfunction and promotes sorafenib resistance [181].

### Crosstalk between glucose and amino acid metabolism

Similarly, in the metabolic reprogramming of HCC, glucose metabolism also plays a pivotal role in regulating amino acid metabolism through three key molecular mechanisms: (i) In HCC, the glycolytic key enzyme GAPDH can directly bind to the promoter region of the PHGDH gene, promoting its transcription and thereby upregulating serine biosynthesis. This mechanism directly links glucose metabolic status with the serine metabolic pathway, driving one-carbon metabolism, histone methylation, and HCC progression [182]. In addition, GAPDH can regulate the activity of serine racemase (SRR), indirectly controlling the production of D-serine, further reflecting the ability of glucose metabolism to reshape the amino acid metabolic network through enzymatic activity modulation [183]. (ii) Glucose metabolic levels can activate mTORC1 through upstream nutrient-sensing signals, thereby affecting the expression of amino acid transporters (such as SLC1A5 and SLC7A5) on the cell membrane. Under high-glucose conditions, activated mTORC1 enhances the uptake of amino acids—particularly key ones like leucine and glutamine—facilitating protein synthesis and supporting cell growth and proliferation [184]. Thus, glucose metabolism indirectly determines cellular amino acid dependence and uptake strategies, forming a coordinated glucose–amino acid metabolic network. (iii) When glucose metabolism is active, pyruvate is often prevented from entering the TCA cycle by PDK1, reducing mitochondrial oxidation of glucose-derived carbon. Under such conditions, cells frequently compensate by enhancing glutaminolysis to replenish TCA cycle intermediates [185]. In sorafenib-resistant HCC cells, upregulated glucose metabolism increases PPAR-δ expression, which in turn elevates PDK1 and glutaminase GLS1 levels, promoting the conversion of glutamine to α-ketoglutarate to sustain TCA cycle function and cell survival [186]. This metabolic compensation mechanism demonstrates how glucose metabolism indirectly enhances amino acid dependency by regulating mitochondrial carbon flux.

On the other hand, amino acid metabolism reciprocally influences glucose metabolism through the activity of glutamate-pyruvate transaminase (GPT/ALT), which catalyzes the conversion of alanine to pyruvate. In HCC cells, GPT overexpression increases ATP production and induces GLUT1 expression, promoting HCC progression in a time-dependent manner and providing a vital energy source for HCC growth and proliferation [187].

### Crosstalk between lipid and amino acid metabolism

In the metabolic reprogramming of HCC, lipid metabolism plays a pivotal role in regulating amino acid metabolism through intricate crosstalk. A key mechanism involves CPT1A, the rate-limiting enzyme of FA oxidation, which is significantly downregulated in HCC. The genetic ablation of CPT1A disrupts lipid metabolism and reduces acetyl-CoA production, leading to the accumulation of BCAAs. This metabolic shift results in the hyperactivation of the mTOR signaling pathway, which is strongly associated with poor prognosis in HCC [188].

On the other hand, amino acid metabolism also modulates lipid metabolism through two distinct molecular mechanisms: (i) The downregulation of oxoglutarate dehydrogenase-like (OGDHL) suppresses the activity of the α-ketoglutarate dehydrogenase complex in HCC cells, thereby impairing mitochondrial glucose oxidation, promoting glutamine consumption, and enhancing the antioxidant defense system of HCC cells, ultimately leading to mitochondrial dysfunction. Moreover, OGDHL silencing activates the mTORC1 and SREBP pathways in an α-ketoglutarate-dependent manner, promoting FA synthesis and driving tumor proliferation in HCC patients [189]. Similarly, the loss of the serine transporter SLC25A15 results in ammonia accumulation, which inhibits OGDHL expression and further enhances oncogenic processes through this metabolic axis [190]. (ii) The overexpression of suppressor of cytokine signaling 5 (SOCS5) inhibits PI3K/Akt/mTOR-mediated autophagy via the transcription of BP1, which drives glutamine-derived de novo lipogenesis and promotes HCC metastasis [191].

## Precision diagnosis and treatment based on metabolic reprogramming in HCC

Surgery, radiotherapy, and chemotherapy remain the cornerstone treatments for HCC and continue to play a crucial role in clinical practice. Surgical resection and liver transplantation are widely accepted as the most effective approaches for early-stage HCC; however, the risk of recurrence due to microvascular invasion (MVI) remains a major limitation of postoperative outcomes. Despite rigorous screening, recent studies indicate that MVI still results in a recurrence rate of 30%–50% [192]. Radiotherapy and chemotherapy are often hampered by widespread drug resistance and severe side effects. In recent years, the rapid development of immunotherapy has improved outcomes for patients with advanced HCC to some extent, yet challenges such as immune-related adverse events and individual variability persist. The metabolic reprogramming of HCC cells enables them to adapt to the demands of rapid proliferation, and the in-depth investigation of these metabolic alterations has provided critical insights into the discovery of novel diagnostic markers and therapeutic targets. These findings have paved the way for the development of precision diagnosis and treatment strategies. In this review, we summarize the latest advancements in precision diagnostics and therapeutic outcomes based on the reprogramming of glucose, lipid, and amino acid metabolism (Table 1).

**Table 1:** The strengths and weaknesses of the different approaches to reverse HCC therapy resistance and future directions

Approaches	Strengths	Weaknesses	Future directions
Chemotherapy	1) Non-aetiology-specific medications, such as statins and metformin, are inexpensive; 2) Have a favourable adverse effect profile; 3) Have several extrahepatic metabolic benefits.	1) Unpredictable severe side effects development; 2) Development of resistance; 3) Concerns regarding their cost-effectiveness; 4) Variable radiological response; 5) Variable patient outcome.	1) Harnessing big ‘omics’ data and AI for drug discovery in HCC; 2) Identify an enriched patient population at high risk of HCC, and use molecular markers of early HCC or perhaps pre-cancerous lesions as end points.
Targeted therapy	1) Provide some benefit in an adjuvant setting in patients with locally advanced HCC to prevent or delay relapse; 2) The next generation sequencing has encouraged the development of dual targets.	1) Which targeted agent is the most appropriate for a specific HCC patient cohort is still not clear; 2) The majority of patients have to take into account the economic consequences; 3) Intratumorally heterogeneity.	1) Discovery and validation of novel biomarkers to reliably predict the response to treatment; 2) Improving response rates and limiting avoidable toxicity in those who are unlikely to benefit.
Immunotherapy	1) The combination of multikinase inhibitors with ICIs has been tested in untreated unresectable HCC, with robust antitumor activity [227]; 2) Combinations of different ICIs present has also been examined in a phase I/II trial involving patients with advanced-stage HCC (NCT04039607); 3) Long-term curative effect may be better.	1) Few studies have associated immune classes, gene signatures and even specific mutations; 2) Immune-related adverse events are a multi-system class effect; 3) Some of advanced-stage HCC patients will not be eligible for the new standard-of-care owing to autoimmune diseases and prior liver transplantation; 4) The evidence of using ICIs in the neoadjuvant setting is lacking.	1) Future studies will have to address whether new ICI-based combinations can overcome resistance to treatment with atezolizumab and bevacizumab in the first-line setting; 2) The first-line treatment landscape of the future will probably include a triplet combination or a biomarker-based regimen enabling population enrichment.
Epigenetic modification inhibitors	1) It can simultaneously affect a variety of epigenetic modification enzymes (such as DNA methyltransferase, histone deacetylase, histone methyltransferase, etc.); 2) The antitumor activity of immune cells can be enhanced by regulating the tumor microenvironment; it can reverse the drug resistance of HCC cells to traditional chemotherapy and targeted therapy.	1) There are great differences in the efficacy of HCC treatment, and some patients may be ineffective or resistant; 2) There are significant differences in curative effect among different patients, which may be related to individual epigenetic characteristics, tumor heterogeneity and other factors; 3) Long-term use may lead to drug resistance and recurrence of HCC cells.	1) Through the analysis of the epigenetic regulatory network, the key epigenetic factors related to drug resistance were identified, and inhibitors for these factors were designed. 2) Real-time monitoring of changes in epigenetic status supports dynamic adjustment during treatment. 3) Develop an individualized treatment plan based on the epigenetic characteristics of patients, and improve the treatment effect through precision medicine.
Gene therapy	1) Allow local production of the cytokine at the tumor site, thus achieving high intratumor or peritumoral levels with low serum concentration; 2) It can play a role through a variety of mechanisms, including inducing HCC cells apoptosis and inhibiting tumor angiogenesis; 3) Individual designs can be made according to the genome characteristics of patients.	1) The lack of efficacy likely depends on short duration of transgene expression; 2) The HCC tissue is not easily infected with adenoviral vectors; 3) Infect peritumoral tissue with long-term expression vectors encoding cytokines, which may be potentially toxic.	1) The development of vectors with high transduction efficiency, high transgene capacity, and acceptable toxicity profile; 2) Identification of the ideal therapeutic gene or gene combinations for each therapeutic indication; 3) Development of systems allowing desired duration and regulation of the gene expression.

AI, artificial intelligence; HCC, hepatocellular carcinoma; ICIs, immune checkpoint inhibitors

### Precision medicine based on reprogramming glucose metabolism

With advancements in metabolomics and molecular imaging technologies, glucose metabolism-based precision medicine has become a cornerstone in the early screening, staging, and therapeutic monitoring of HCC. The reprogramming of glucose metabolism in HCC often involves the dysregulated expression or mutation of genes related to glycolysis and the PPP, such as GLUT, PKM2, LDHA, FOXC2, HOXB6, and TBL1XR1. Specific mutations or copy number variations in these metabolic genes, detected in circulating tumor DNA (ctDNA), serve as potential biomarkers reflecting the metabolic state of HCC cells, offering insights for early diagnosis and personalized treatment.

High glucose uptake by HCC tissues also makes β-2-[^18^F] fluoro-2-deoxy-D-glucose positron emission tomography/computed tomography (^18^F-FDG PET/CT) a widely used tool for HCC diagnosis and disease monitoring. By tracing glucose metabolism products like ^18^F-FDG, PET/CT provides a clear depiction of the macrotrabecular massive (MTM) subtype, achieving an area under the receiver operating characteristic curve (AUC) of 80.6%, with 78.9% sensitivity and specificity. This underscores its robust performance in early detection, staging, and treatment response evaluation [193]. Moreover, MVI is a well-established predictor of poor differentiation and unfavorable prognosis, frequently involves genes linked to glucose metabolism. Radiomics approaches using preoperative multiphase CT imaging facilitate MVI prediction, aiding in the identification of patients with adverse histological profiles and enabling more tailored treatment strategies [194].

In terms of precision therapy, metformin, a first-line antidiabetic drug, has garnered attention for its tumor-suppressive effects mediated by AMPK activation and mTORC1 inhibition, thereby reducing glucose uptake and utilization in HCC cells. A recent multicenter retrospective cohort study demonstrated significantly prolonged median OS (mOS) and median PFS (mPFS) in type II diabetic HCC patients treated with metformin alongside TACE compared to non-metformin users. The metformin group also exhibited lower risks of HCC progression and cancer-specific mortality [195]. Furthermore, the aberrant activation of the PI3K/Akt/mTOR signaling pathway in HCC enhances glucose transport and glycolysis, making it a promising therapeutic target. Ongoing clinical trials are evaluating the efficacy of PI3K/Akt/mTOR inhibitors in HCC treatment. A recent systematic review and meta-analysis revealed that immunosuppressive regimens incorporating Sirolimus or Everolimus post-liver transplantation significantly improved OS and recurrence-free survival (RFS) in HCC patients, with a reduced risk of nephrotoxicity compared to non-mTOR-based therapies [196].

### Precision medicine based on reprogramming lipid metabolism

Mass spectrometry (MS), renowned for its high sensitivity and resolution, has become an indispensable tool for the qualitative and quantitative analysis of lipid metabolites in the blood, urine, and tissues of HCC patients. Elevated levels of lipid metabolites, such as FAs, triglycerides, and phospholipids, along with the overexpression of lipid metabolism-related enzymes like fatty acid synthase and phosphatidylcholine synthase, have been identified as potential biomarkers for the early diagnosis of HCC. High-throughput multiple reaction monitoring mass spectrometry (HST-MRM-MS), a novel quantitative method based on MS technology, has demonstrated high accuracy in the absolute quantification of serum biomarkers in HCC patients [197].

With the advancement of magnetic resonance imaging (MRI) and proton magnetic resonance spectroscopy (MRS), imaging techniques based on lipid metabolism have been increasingly integrated into the precise diagnosis of HCC. A metabolic analysis of plasma and serum using ^1^H nuclear magnetic resonance (NMR) in Nigerian and Egyptian cohorts revealed significant differences in the levels of LDL, VLDL, and lactate between HCC patients and healthy volunteers [198]. Additionally, a study assessing intratumoral fat content using preoperative enhanced MRI in patients undergoing solitary HCC resection demonstrated that homogeneous intratumoral fat was associated with prolonged RFS and OS in the Asian cohort, though no significant improvement was observed in the European cohort [199].

In the realm of precision therapy, statins, widely known as HMG-CoA reductase inhibitors, have garnered attention not only for their cholesterol-lowering effects but also for their emerging antitumor potential. Two recent meta-analyses reported a 43% reduction in HCC risk associated with statin use (including atorvastatin, rosuvastatin, fluvastatin, lovastatin, pravastatin, simvastatin, pitavastatin, and cerivastatin) compared to non-use [200], along with a significant decrease in all-cause mortality among HCC patients [201]. Subgroup analysis further revealed that statin use was associated with reduced mortality in patients with stage I–III HCC and those receiving palliative treatment, but this effect was not observed in stage IV HCC patients or those who had undergone curative treatment [201].

### Precision medicine based on reprogramming amino acid metabolism

HCC cells actively reprogram amino acid metabolism to sustain energy production, bolster antioxidant defense, and evade immune surveillance, making amino acid-related metabolites critical biomarkers for precise diagnosis. In clinical practice, glutaminase (GLS) has been adopted as a prognostic marker, with alterations in its expression profile serving as a predictor of postoperative survival in HCC patients [202]. High-performance liquid chromatography-mass spectrometry (HPLC-MS), which integrates high chromatographic separation efficiency with the sensitivity and resolution of MS, enables early screening of HCC by detecting glutamine levels and related enzymatic activity in blood samples. Using HPLC-MS for untargeted metabolomics, the researchers demonstrated significantly elevated levels of DL-3-phenyllactic acid, L-tryptophan, glycocholic acid, and 1-methylnicotinamide in the portal venous serum and HCC tissues of patients compared to healthy controls, all of which were associated with reduced postoperative survival [203]. Furthermore, combining glutamine metabolic profiling with dynamic CT or contrast-enhanced MRI has been shown to effectively evaluate HCC differentiation [204].

In addition to glutamine metabolism-related markers, HCC cells enhance their proliferative and antioxidative capacities by activating the serine-glycine synthesis pathway, which facilitates nucleotide and glutathione production. Consequently, untargeted metabolomic analysis of serine and glycine profiles, alongside immunohistochemical assessment of PHGDH expression in HCC tissues, presents a promising approach for targeted HCC therapy [205]. Notably, HCC patients frequently exhibit dysregulated BCAA metabolism, which plays a crucial role in HCC energy homeostasis and signaling pathways. Metabolomic analysis of BCAA distribution in the blood or urine can establish quantitative diagnostic standards. However, a recent randomized clinical trial evaluating the impact of BCAA supplementation on postoperative HCC recurrence found no significant difference in RFS and OS between the control group (surgical treatment alone) and the BCAA-supplemented group. Interestingly, BCAA supplementation showed potential benefits in younger patients with mild glucose intolerance [206]. Further research is needed to clarify the long-term impact of BCAA supplementation on recurrence risk following curative resection of HCC.

In the realm of precision therapy, targeting glutamine metabolism has emerged as a compelling strategy. GLS inhibitors such as 968 (5-(3-Bromo-4-(dimethylamino)phenyl)-2,2-dimethyl-2,3,5,6-tetrahydrobenzo[a]phenanthridin-4(1 H)-one) [207], BPTES (bis-2-(5-phenylacetamido-1,3,4-thiadiazol-2-yl) ethyl sulphide) [189], and CB-839 [113] have been shown to reduce glutamine consumption and nucleotide synthesis in HCC cells, thereby suppressing proliferation, shrinking xenograft tumors, and enhancing sensitivity to sorafenib. Furthermore, amino acid transporters such as SLC7A11 and SLC1A5 play essential roles in supporting HCC proliferation, metabolic reprogramming, antioxidant defense, and immune evasion. Modulating the PTM of SLC7A11—via Activating Transcription Factor 4 (ATF4) ablation [66], USP8 inhibition with DUB-IN-3 [119], or the use of ferroptosis modulator DAZAP1 [208]—sensitises HCC cells to ER stress-induced cell death, significantly reducing the GSH/GSSG ratio and increasing lipid peroxidation, thereby effectively inhibiting proliferation and colony formation. Moreover, discoid protein domain receptor 1 (DDR1) interacts with SLC1A5 to activate the mTORC1 signaling pathway, promoting HCC proliferation and correlating with poor patient prognosis [209]. Inhibition of DDR1-mediated SLC1A5 stabilization with the proteasome inhibitor ammonium chloride effectively attenuates this oncogenic effect, highlighting its potential as a therapeutic target [209]. These amino acid metabolism-based strategies remain in preclinical stages, further clinical investigations are essential to establish their safety and efficacy.

### Combined immunotherapy / chemotherapy / radiotherapy / artificial intelligence (AI)

Besides the use of metabolic-targeting agents alone to inhibit the reprogramming of glucose, lipid, and amino acid metabolism in HCC cells, combining these agents with immunotherapy, chemotherapy, or radiotherapy offers a promising approach to integrated diagnosis and treatment. This combination strategy not only enhances therapeutic efficacy but also broadens opportunities for personalized treatment. Furthermore, the application of AI as a supportive tool enables more efficient prediction of complications and identification of diagnostic biomarkers and uncovers novel therapeutic targets for HCC. However, the development of drugs targeting HCC metabolic pathways—such as inhibitors of glucose, lipid, and amino acid metabolism—is still in its early stages in HCC treatment. Therefore, Table 2 summarizes recent advances in the combined use of metabolic-targeting agents and immunotherapy, chemotherapy, or radiotherapy in HCC.

**Table 2:** The combination therapy drugs targeting glucose metabolism, lipid metabolism and amino acid metabolism reprogramming of HCC

Approaches	Metabolic type	Targets	Compounds	Combination therapies	Mechanisms of action	Highest phase and Results reporting dates	NCT No.	Years and References
Combined immunotherapy	Glucose metabolism	GLUT1	Salvianolic acid B	PD-1 inhibitor	Downregulates HIF-1α, therefore inactivating GLUT1.	Pre-clinical	\	2024, [228]
	Glucose metabolism	GLUT1	Sirolimus	Huai Er	Downregulate the expression of HIF-1α induced by hypoxia and weaken its mediated glycolytic effect.	Pre-clinical	\	2022, [229]
	Lipid metabolism	JAK2/STAT3 pathway	TNKS1BP1	PD-1 inhibitor	Increasing infiltration of tumor-infiltrating lymphocytes as well as augmenting the effect of cytotoxic T lymphocytes.	Pre-clinical	\	2024, [230]
	Amino acid metabolism	Glutamine	DRP-104	Durvalumab	Glutamine antagonist DRP-104 in combination with durvalumab in patients with advanced-stage fibrolamellar HCC.	Phase Ib/II trial (Recruiting)	NCT06027086	2024, [231]
	Amino acid metabolism	ASS1	ADI-PEG 20	Cisplatin	Restored ASS1 protein levels in most of the cell lines studied.	Phase I trial in 2020	NCT02029690	2014, [232]
	Amino acid metabolism	Phosphatidylserine	Bavituximab	Pembrolizumab	Targeting membranous phosphatidylserine may induce pro-inflammatory and -immune stimulating effects that enhance immunotherapy activity.	Phase II trial in 2024	NCT03519997	2024, [214]
Combined chemotherapy	Glucose metabolism	G6PD	6AN	Regorafenib	NADPH and NADPH/NADP^+^ and increased ROS levels inhibited HCC proliferation.	Pre-clinical	\	2023, [233]
	Glucose metabolism	PDH, PC	CPI-613, CHCA	Glutamine restriction dietary intervention	PDH or PC inhibitors further disrupt the metabolic rewiring of the TCA cycle induced by dietary glutamine depletion in HCC.	Pre-clinical	\	2022, [234]
	Glucose metabolism	PDHB	HY-N0022 (Isoacteoside)	Sorafenib	PDHB knockout enhanced the expression of PCK1 and inhibited the glycolysis process.	Pre-clinical	\	2025, [32]
	Glucose metabolism	PI3K/AKT/mTOR pathway	Arenobufagin	Rapamycin	Inhibited poly(ADP-ribose) polymerase cleavage, light chain 3-II activation and mTOR.	Pre-clinical	\	2013, [235]
	Glucose metabolism	mTOR pathway	Temsirolimus	Vinblastine	Specific and concerted down-regulation of several key anti-apoptotic/survival proteins (survivin, Bcl-2, and Mcl-1).	Pre-clinical	\	2012, [236]
	Glucose metabolism	FZD10/β-catenin/c-Jun/MEK/ERK pathway	FH535	Lenvatinib	By inhibiting β-catenin and YAP1, the self-renewal, tumorigenicity and metastasis of liver CSCs were inhibited.	Pre-clinical	\	2023, [237]
	Lipid metabolism	Nrf2/GSR	Carmustine	Sorafenib	Inhibition of GSR strengthens the efficacy of sorafenib through GSH depletion and the accumulation of lipid peroxide products in SLC27A5-knockout and sorafenib-resistant HCC cells.	Pre-clinical	\	2023, [71]
	Lipid metabolism	FASN	Cerulenin	Paclitaxel	The resistant cells could be resensitized to these different classes of drugs by silencing the protein levels of P-gp, Cav-1 and FASN.	Pre-clinical	\	2013, [218]
	Lipid metabolism	EGFR-STAT3-ABCB1 pathway	Stattic	Lenvatinib	Directly targeting the downstream effector ABCB1 of STAT3, thus weakening the exocytosis of HCC cells and significantly promoting the apoptosis of HCC cells.	Pre-clinical	\	2022, [238]
	Amino acid metabolism	Glutaminase	CB-839	Standard chemotherapy	Synergistically kills HCC cells by dual inhibition of glutamine metabolism, depleting glutathione, and inducing excessive ROS and DNA damage.	Phase I trial in 2019	NCT02071862	2019, [239]
	Amino acid metabolism	SLC1A5	SLC1A5 knockdown	TACE	SLC1A5 is positively correlated with hypoxia, angiogenesis, and immunosuppression.	Pre-clinical	\	2024, [240]
	Amino acid metabolism	ASCT2	Resveratrol	Cisplatin	Increased ROS production, γH2AX foci formation and apoptosis in HCC cells.	Pre-clinical	\	2018, [217]
	Amino acid metabolism	GLS2	MSO	Crisantaspase	Crisantaspase and MSO depleted serum glutamine, lowered glutamine in HCC tissue, and inhibited liver GS activity.	Pre-clinical	\	2014, [241]
	Amino acid metabolism	PHGDH	NCT-503	Sorafenib	Inactivation of PHGDH elevates ROS level and induces HCC apoptosis upon sorafenib treatment.	Pre-clinical	\	2019, [242]
	Amino acid metabolism	Akt/mTOR/eIF4E pathway	Ribavirin	Doxorubicin	Targeting eIF4E by ribavirin sensitizes HCC cell response to doxorubicin.	Pre-clinical	\	2018, [243]
	Amino acid metabolism	mTOR pathway	2a	Radiotherapy	Compound 2a, with a 4,7-dihydro-[1,2,4]triazolo[1,5-a]pyrimidine scaffold, exhibited promising potency against mTOR.	Pre-clinical	\	2020, [244]
Combined radiotherapy	Amino acid metabolism	CPS1	10,074-G5	Radiotherapy	Inactivation of c-Myc using 10,074-G5, a specific c-Myc inhibitor, could partially attenuate the proliferation and radioresistance induced by depletion of CPS1.	Pre-clinical	\	2023, [245]

mTORC1, mammalian target of rapamycin complex 1; AMPK, adenosine 5’-monophosphate (AMP)-activated protein kinase; HK2, hexokinase 2; ROS, reactive oxygen species; FZD10, frizzled homolog 10; YAP1, yes-associated protein 1; Nrf2, nuclear factor erythroid 2-related factor 2; SLC27A5, solute carrier family 27 member 5; GSR, glutathione reductase; FASN, fatty acid synthase; P-gp, p-glycoprotein; Cav-1, caveolin-1; EGFR, epidermal growth factor receptor; STAT3, signal transducer and activator of transcription 3; ABCB1, ATP binding cassette subfamily B member 1; JAK2, janus kinase 2; ASCT2, alanine-serine-cysteine transporter 2; GLS2, glutamine synthase 2; MSO, methionine-L-sulfoximine

#### Combined immunotherapy

Metabolic reprogramming in the microenvironment of HCC not only occurs within HCC cells but also involves metabolic competition and signal interaction with immune cells, which plays a key role in determining treatment response. On the one hand, HCC cells suppress the metabolic activity of CD8⁺ T cells and NK cells—driving them into a state of functional exhaustion—by enhancing glycolysis and lipid synthesis, releasing lactate, and depleting glucose [210]. On the other hand, activated CD8⁺ T cells and NK cells can increase their own glycolytic activity to boost IFN-γ production, thereby promoting HCC antigen presentation and apoptosis. NK cells also maintain sustained activity through fatty acid oxidation, enhancing their HCC recognition and cytotoxic capacity [210]. In addition, tumor-associated macrophages (TAMs) tend to polarize toward an M2 phenotype under tumor-driven metabolic conditions, which suppresses T cell function; however, under specific metabolic environments, TAMs can be reprogrammed into an M1 phenotype, releasing ROS and TNF-α to counteract HCC growth [211, 212]. These bidirectional metabolic regulatory mechanisms suggest that targeting immune metabolic states may synergistically enhance the efficacy of existing immunotherapies.

As mentioned earlier, Serine and Arginine Rich Splicing Factor 10 (SRSF10) is a crucial gene implicated in resistance to programmed cell death protein 1 (PD-1) therapy and the immunosuppressive microenvironment of HCC. In advanced, unresectable HCC, the researchers conducted a preclinical study using various HCC-bearing mouse models and patient-derived organoids to explore the biological role of SRSF10 in immune evasion [213]. Flow cytometry analysis revealed that the combination of the selective SRSF10 inhibitor 1C8 and PD-1 monoclonal antibody (mAb) significantly increased the population of cytotoxic CD8⁺ T cells and reduced the proportion of F4/80^+^ and CD206^+^ macrophages, which promote HCC growth, compared with either treatment alone. Notably, no significant toxicity, including weight loss, was observed in the treated mice. The results were further validated using three publicly available immunotherapy datasets, demonstrating that patients with lower SRSF10 expression were associated with significantly longer OS. These findings suggest that the combination of 1C8 and PD-1 mAb enhances antitumor efficacy in both mouse and human preclinical HCC models and may represent a promising strategy to restore the immune system’s intrinsic antitumor capabilities in HCC patients. Clinically, SRSF10 may also serve as a biomarker for predicting resistance to immunotherapy across various solid tumors [213].

Furthermore, a phase 2 clinical trial evaluated the hypothesis that targeting membrane phosphatidylserine could enhance the efficacy of immunotherapy [214]. Among 28 evaluable patients, the combination of bavituximab, a phosphatidylserine-targeting antibody, and pembrolizumab achieved an objective response rate of 32.1%, meeting the pre-specified endpoint. The mPFS was 6.3 months. These results indicate that targeting phosphatidylserine may exert a synergistic effect with PD-1 immune checkpoint inhibitors without increasing toxicity. Future studies on this therapeutic strategy should focus on identifying biomarkers that characterize the tumor microenvironment prior to treatment [214].

#### Combined chemotherapy

In glucose metabolism, 6-phosphogluconate dehydrogenase (6PGD), a key enzyme in the pentose phosphate pathway, is upregulated in HCC tissues. The researchers demonstrated that the combination of chemotherapy and the 6PGD inhibitor physcion achieved superior efficacy in suppressing HCC growth and survival compared with chemotherapy alone, which was mechanistically linked to the activation of AMPK and ACC1 [215]. Additionally, dichloroacetate (DCA), a small-molecule metabolic modulator, has been shown to reverse the glycolytic phenotype by inhibiting HK, a rate-limiting enzyme in glycolysis. This reversal induces ROS production and triggers apoptosis in various tumor cells. ADM, an anthracycline-based chemotherapeutic agent, inhibits HCC growth by disrupting DNA replication and cell division. The above researchers further evaluated whether DCA could potentiate the antitumor efficacy of ADM in HCC cell lines (HCC-LM3 and SMMC-7721) [216]. Their results demonstrated that the combination of DCA and ADM significantly decreased cell viability, increased the proportion of apoptotic cells, and elevated intracellular ROS levels compared with either agent alone. Notably, this combination did not result in increased cytotoxicity in normal liver cells.

In amino acid metabolism, the above researchers also reported that L-buthionine-S, R-sulfoximine (BSO), a glutathione synthase inhibitor, enhanced the sensitivity of HCC cells to the DCA-ADM combination therapy [216]. However, the underlying molecular mechanism remains to be elucidated. Moreover, as mentioned earlier, ASCT2, a key glutamine transporter on the HCC cell membrane, facilitates the uptake of extracellular glutamine into HCC cells. Intracellular glutamine is subsequently metabolized to produce glutathione, which enhances antioxidant capacity and contributes to chemoresistance against agents like cisplatin. Resveratrol, by downregulating ASCT2 expression and thereby reducing glutamine and glutathione uptake, significantly increased ROS levels in HCC cells when combined with cisplatin. This combination markedly enhanced the sensitivity of HCC cells to cisplatin treatment [217].

In lipid metabolism, the expression of FASN increases in a stage-dependent manner and is associated with poor survival outcomes in HCC patients. The investigators investigated the molecular mechanisms underlying the intrinsic and acquired resistance of HCC to paclitaxel and identified a strong association between FASN expression and both resistance phenotypes [218]. Downregulation of FASN through siRNA and/or specific pharmacological inhibitors restored the sensitivity of HCC cells to paclitaxel. Another crucial enzyme in lipid metabolism, acyl-CoA synthetase long-chain family member 4 (ACSL4), plays a rate-limiting role in fatty acid synthesis. ACSL4 modulates de novo lipogenesis in HCC cells by upregulating SREBP1 and its downstream lipogenic enzymes via the c-Myc pathway [64]. The investigators evaluated the prognostic significance of ACSL4 in postoperative adjuvant TACE (PA-TACE) and found that high ACSL4 expression was associated with increased recurrence risk following PA-TACE. Canagliflozin, a clinically approved drug for type 2 diabetes, mimics glucose starvation and selectively inhibits the growth of ACSL4-low xenograft HCC [219].

#### Combined radiotherapy

HCC cells often exhibit dysregulated expression of BCAT or BCKDH, leading to enhanced metabolic reprogramming of BCAAs. This metabolic shift redirects carbon and nitrogen from BCAAs into the nucleotide synthesis pathway and supports HCC cell proliferation through the production of metabolic intermediates. In the context of radiofrequency ablation (RFA) therapy, a randomized trial assessed liver function and HCC recurrence rates in patients undergoing RFA [220]. The study demonstrated that patients receiving oral supplementation with Aminoleban EN, a BCAA-enriched nutritional formula, exhibited significantly longer event-free survival (EFS) and lower intrahepatic recurrence rates compared with those on a standard post-RFA diet (control group). Notably, only the BCAA group showed significant improvement in the Short Form-8 mental component score, suggesting enhanced psychological well-being. These findings indicate that the combination of RFA and oral BCAA supplementation may help alleviate psychological stress while reducing the risk of intrahepatic recurrence and associated complications in HCC patients. Another prospective controlled trial further evaluated liver function and nutritional status in HCC patients following RFA. The results revealed that patients in the BCAA group demonstrated significantly higher scores in baseline health status, physical functioning, and social functioning, alongside a marked improvement in the non-protein respiratory quotient [221]. These findings underscore the potential of BCAA supplementation to improve both physical and nutritional status in HCC patients undergoing RFA therapy.

#### Combined artificial intelligence (AI)

HCC is a common complication of liver cirrhosis, often resulting from long-term asymptomatic chronic inflammation and hepatic regeneration, leading to late-stage diagnosis. Recently, the researchers developed a plasma metabolomics-based scoring tool using a machine learning framework to predict cirrhosis-related complications [222]. This model, which analyzed metabolomic states derived from 168 circulating metabolites, demonstrated high accuracy in predicting the 10-year risk of cirrhosis complications in the training cohort (time-dependent AUC: 0.84). Notably, it outperformed both the fibrosis-4 index (AUC difference: 0.06 [0.03–0.10]) and the polygenic risk score (0.25 [0.21–0.29]). Despite advances in HCC management, effective early diagnostic biomarkers remain lacking. Using a dynamic network construction algorithm called the early warning signal data-driven approach (EWS-DDA) can identify key genes and metabolites involved in fatty acid metabolism [223]. This analysis revealed three significant gene ratios—PLA2G4F/PPARGC1A and ACOT6/HMGCLL1 (both upregulated) and CYP2C8/SCP2 (downregulated)—as well as eight metabolite ratios, including several lysophosphatidylcholine (LPC) to free FA (FFA) and LPC to triacylglycerol (TAG) ratios. These findings suggest their potential as biomarkers for HCC clinical management.

Looking ahead, integrating AI with multi-omics data offers promising opportunities for novel therapeutic discoveries in HCC. As previously mentioned, GLUT1 (also known as SLC2A1) is a unidirectional glucose transporter that facilitates glucose uptake across the mammalian plasma membrane. By integrating a combination of multiple machine learning algorithms, it is possible to develop a novel, stable, and highly accurate machine learning-based prognostic signature (MLPS) [224]. SLC2A1, identified as the core gene of MLPS, was highly expressed in advanced-stage HCC, and its downregulation significantly suppressed the malignant behavior of HCC cells. Moreover, SLC2A1 expression was associated with increased sensitivity to dasatinib and vincristine, highlighting its potential as a therapeutic target. Together, MLPS and SLC2A1 provide valuable tools for HCC prognosis and individualized targeted therapy [224].

## Conclusion and future perspectives

Primary liver cancer remains one of the leading causes of cancer-related mortality worldwide, with HCC being its predominant form. Metabolic reprogramming has emerged as a hallmark of HCC progression, driving HCC proliferation, survival, and immune evasion. Recent studies have demonstrated that alterations in glucose, lipid, and amino acid metabolism, together with signals from the tumor microenvironment, form a highly complex regulatory network that hinders the translation of basic discoveries into clinical applications.

Future research should prioritize elucidating the molecular mechanisms and regulatory networks of HCC metabolic reprogramming. Advanced technologies such as high-throughput sequencing, metabolomics, and proteomics provide powerful tools to characterize the dynamic metabolic changes across different stages of HCC. Particular attention should be paid to the interplay between metabolic reprogramming and immune responses, as targeting key metabolic pathways may enhance the efficacy of immunotherapy and support the development of metabolism-based immunotherapeutic strategies. Moreover, integrating virus-associated metabolic signatures, cross-organ interactions such as the spleen–liver and gut–liver axes, and multi-omics approaches may improve patient stratification and inform precision therapeutic strategies. Our group’s work has further highlighted the systemic role of the spleen in promoting liver fibrosis and HCC progression through immune regulation, reinforcing the importance of considering extrahepatic influences in HCC pathogenesis [225, 226]. Equally critical is the rigorous validation of potential biomarkers and therapeutic targets, together with the acceleration of their translational application through innovative targeted therapies and synergistic treatment strategies. Interdisciplinary collaboration across oncology, metabolism, immunology, and bioinformatics, combined with the application of emerging technologies such as artificial intelligence and big data analytics, will be vital in advancing these goals. Nevertheless, substantial challenges remain. The pronounced heterogeneity of HCC, the dynamic interactions among tumor, immune, and metabolic pathways, and the limited availability of clinically validated biomarkers restrict the immediate clinical utility of current insights. More comprehensive and integrative studies are needed to bridge the gap between experimental discoveries and practical applications in diagnosis and therapy.

In conclusion, although the field of HCC metabolic reprogramming faces significant obstacles, it also offers unprecedented opportunities. A more integrated and collaborative research framework—encompassing mechanistic studies, biomarker validation, and translational innovation—will be essential to transform current knowledge into effective diagnostic and therapeutic strategies, ultimately improving patient outcomes.

## Data Availability

No datasets were generated or analysed during the current study.

## Abbreviations

HCC: Hepatocellular carcinoma
PI3K/AKT/mTOR: Phosphoinositide 3-kinase / Protein kinase B / Mammalian target of rapamycin
HK: Hexokinase
PFK: Phosphofructokinase
PKM: Pyruvate kinase M2
PD-L1: Programmed death-ligand 1
TCA: Tricarboxylic Acid Cycle
HIF-1α: Hypoxia-inducible factor 1 alpha
PPP: Pentose phosphate pathway
NADPH: Nicotinamide adenine dinucleotide phosphate
G6PD: Glucose-6-phosphate dehydrogenase
PCK: Phosphoenolpyruvate carboxykinase
INSIG: Insulin-induced gene protein
FBP1: Fructose-1,6-bisphosphatase 1
FA: Fatty acid
FASN: Fatty acid synthase
ACC1: Acetyl-CoA carboxylase 1
ACSL4: Acyl-CoA synthetase long-chain family member 4
NAFLD: Non-alcoholic fatty liver disease
CPT: Carnitine palmitoyltransferase
ACADM/MCAD: Medium-chain Acyl-CoA dehydrogenase
LCAD: Long-chain Acyl-CoA dehydrogenase
PGC-1: Peroxisome proliferator-activated receptor gamma coactivator 1
SIRT1: Sirtuin 1
SphK: Sphingosine Kinase
CPS1: Carbamoyl-phosphate synthetase 1
GSH: Glutathione
GS: Glutamine synthetase
GLS: Glutaminase
USP8: Ubiquitin-specific peptidase 8
PSTK: Phosphoserine-tRNA kinase
γ-GCSh: Gamma-glutamylcysteine synthetase heavy chain
BCAA: Branched chain amino acids
BCAT: Branched-chain aminotransferase
BCKDH: Branched-chain α-keto acid dehydrogenase
ASNS: Asparagine synthetase
PHGDH: Phosphoglycerate dehydrogenase
PTM: Post-translational modifications
PYCR1: Pyrroline-5-carboxylate reductase 1
UDP-GlcNAc: Uridine diphosphate N-acetylglucosamine
OGT: O-Linked N-acetylglucosamine transferase
ChREBP: Carbohydrate response element binding protein
CD: Cluster of differentiation
OGDHL: 2-Oxoglutarate dehydrogenase-like
SOCS5: Suppressor of cytokine signaling 5
GLUT: Glucose transporter
6PGD: 6-Phosphogluconate dehydrogenase
DCA: Dichloroacetate
RFA: Radiofrequency ablation
